# Five New Meroterpenoids from the Fruiting Bodies of the Basidiomycete *Clitocybe clavipes* with Cytotoxic Activity

**DOI:** 10.3390/molecules24224015

**Published:** 2019-11-06

**Authors:** Zhaocui Sun, Xudong Xu, Hanqiao Liang, Xinyi Xia, Guoxu Ma, Leiling Shi

**Affiliations:** 1 Key Laboratory of Bioactive Substances and Resource Utilization of Chinese Herbal Medicine, Ministry of Education, Institute of Medicinal Plant Development, Peking Union Medical College and Chinese Academy of Medical Sciences, Beijing 100193, China; 2 Department of Biomedicine, Beijing City University, Beijing 100094, China; 3 Xinjiang Institute of Chinese and Ethnic Medicine, Urumqi 830002, China

**Keywords:** meroterpenoids, *Clitocybe clavipes*, basidiomycete, cytotoxicity

## Abstract

Five new meroterpenoids, clavipols A–B (**1**–**2**) with a 12-membered ether ring and clavilactones G–I (**3**–**5**) having a 10-membered carbocycle connected to a hydroquinone and an α,β-epoxy/unsaturated lactone, were obtained from the fruiting bodies of the basidiomycete *Clitocybe clavipes*. Their structures were determined by comprehensive analysis of their spectroscopic data, and the absolute configuration of **1** was established by quantum chemical calculations of electronic circular dichroism (ECD). All the isolated compounds (**1**–**5**) were tested for their cytotoxic activity against three human tumor cell lines (Hela, SGC-7901, and SHG-44) in vitro after treatment for 48 h. Compound **4** exhibited moderate cytotoxic activity against Hela and SGC-7901 tumor cell lines, with IC_50_ values of 23.5 and 14.5 µM, respectively.

## 1. Introduction

Secondary metabolites from fungi have attracted the attention of chemists, pharmacologists, and biologists because of their unique chemical structures and potential biological activities [1,2,3]. Meroterpenoids are defined as compounds partially derived from terpenoids [4]. In the past decades, fungal meroterpenoids, including pyripyropene A [5], arisugacins [6], and territrems [6], have been reported to have novel and fascinating chemical structures. They not only have diverse structural skeletons but also show biological activity, such as antitumor [7], anti-inflammatory [8], antioxidant [9], antibacterial [10], and antifungal [10] activities. Considering their unique structure and significant biological activities, total synthesis of fungal meroterpenoids has been achieved by synthetic chemists [11,12].

**Figure 1:**
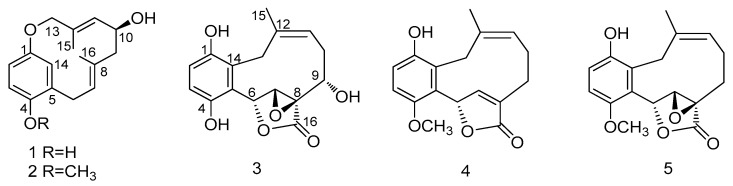
Structures of compounds **1**–**5**.

The fungus *Clitocybe clavipes* has rarely been chemically investigated. To the best of our knowledge, only 10 of its chemical constituents, 5 meroterpenoids, clavilactones A–E, and 5 fatty acid derivatives, have been isolated [13,14,15]. Moreover, the meroterpenoids clavilactones A–E have been reported to have potent pharmacological activity, such as antifungal activity and inhibition of protein tyrosine kinases [13,14]. Especially, clavilactone D was shown to inhibit epidermal growth factor receptor tyrosine kinase, with an IC_50_ value of 5.5 μM [16]. Recently, we have reported the isolation of three novel meroterpenoids from the basidiomycete *C. clavipes*, clavipines A–C, possessing a benzoquinone fused to an azepine ring and a 10-membered carbocycle with α,β-epoxy/unsaturated-ɤ-lactone. [17]. In our ongoing search for structurally unique and biologically valuable metabolites, an investigation of the extracts of the fruiting bodies of the basidiomycete *C. clavipes* led to the isolation of five new meroterpenoids, clavipols A–B (**1**–**2**) and clavilactones G–I (**3**–**5**) (Figure 1). This paper reports the isolation and structural elucidation of the five isolated new meroterpenoids, as well as their cytotoxic activities.

## 2. Results

Compound **1** was isolated as a colorless powder. Its molecular formula was established to be C_16_H_20_O_3_ on the basis of high-resolution electrospray ionization mass spectroscopy (HRESIMS) at *m/z* 259.1329 [M − H]^−^ (calcd for 259.1340), indicating seven degrees of unsaturation. It had an IR absorption band at 3417 cm^−1^, which suggested the presence of hydroxyl groups. The ^1^H-NMR spectrum (Table 1, supplementary Figure S1) of **1** revealed the presence of one ABX aromatic system at [*δ*_H_ 6.60(1H, d, *J* = 8.4 Hz, H-3), 6.56(1H, dd, *J* = 3.0, 8.4 Hz, H-2), 7.23(1H, d, *J* = 3.0 Hz, H-14)], three aliphatic methylenes [*δ*_H_ 3.04(1H, dd, *J* = 7.2, 16.8 Hz, H-6b), 3.34 (1H, dd, *J* = 8.4, 16.8 Hz, H-6a), 2.36 (1H, t, *J* = 12Hz, H-9b), 2.71(1H, dd, *J* = 4.2, 12 Hz, H-9a), 4.46(1H, d, *J* = 12.6Hz, H-13b), 4.64(1H, d, *J* = 12.6 Hz, H-13a)], two olefinic methines [*δ*_H_ 5.74(1H, t, *J* = 7.2Hz, H-7), 5.56(1H, d, *J* = 10.2Hz, H-11)], one oxygenated methine [*δ*_H_ 4.68(1H, m, H-10)], and two methyl groups [*δ*_H_ 1.56(3H, s, H_3_-15), 1.24(3H, s, H_3_-16)]. ^13^C-NMR analysis with the aid of the HSQC spectra of **1** revealed 20 carbon signals composed of two methyls, three methylenes, five olefinic methines, five olefinic quaternary carbons, and one oxygenated methine. The proton signal at *δ*_H_ 4.68 (1H, m, H-10), together with the downfield methine carbon signal at *δ*_C_ 66.0, suggested the existence of a hydroxy group. Comprehensive analysis of 1D-NMR data indicated the existence of a 1,2,4-substituted hydrobenzene unit and the monoterpene moiety.

### Spectra Data were Recorded in CDCl_3_

**Figure 2:**
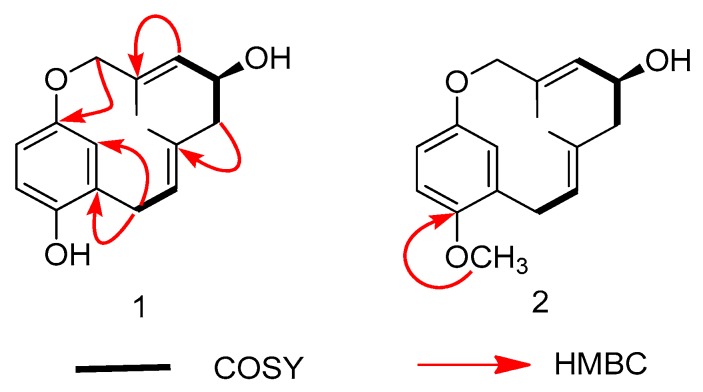
Key ^1^H-^1^H COSY and HMBC correlations for compounds **1**–**2**.

The ^1^H-^1^H COSY spectrum (Figure 2) indicated the presence of two coupling fragments H-6/H-7 and H-9/H-10/H-11. A hydroxy group located at C-10 in **1** was ascertained by the COSY correlations of H-10/H-11 and H-10/H-9, as well as by the HMBC correlations from H-10 to C-9/C-11. The HMBC correlations (Figure 2) from H-6 to C-4/C-5/C-7/C-14 established that the monoterpene was attached to C-5. Meanwhile, the HMBC correlations from H-9 to C-8/C-15 and from H-13 to C-11/C-12/C-13/C-15 indicated compound **1** was an ansa-type monoterpenylbenzenoid with a 12-membered ether ring [18]. The NMR data and biogenetical considerations indicated that the configuration of the two double bonds should be *E*. Finally, the planar structure of **1** was established, as shown in Figure 1, and the compound was given the name clavipol A.

**Figure 3:**
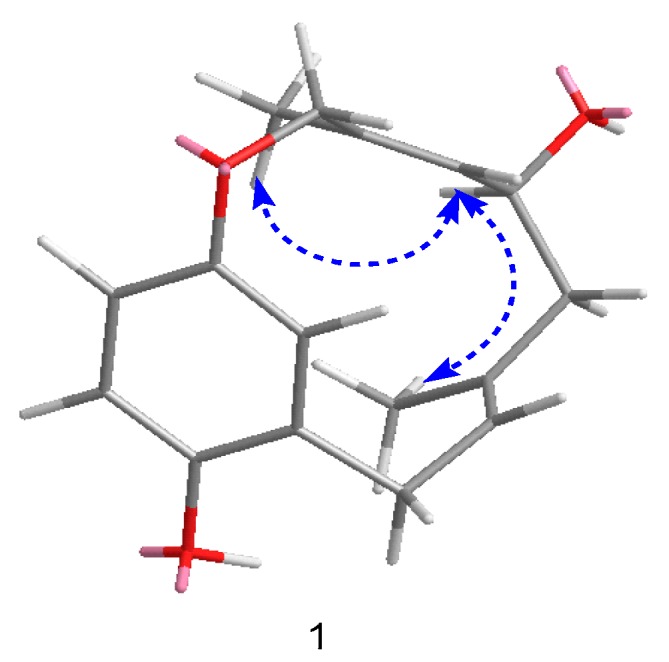
Key rotating overhauser effect correlations for compound **1**.

**Figure 4:**
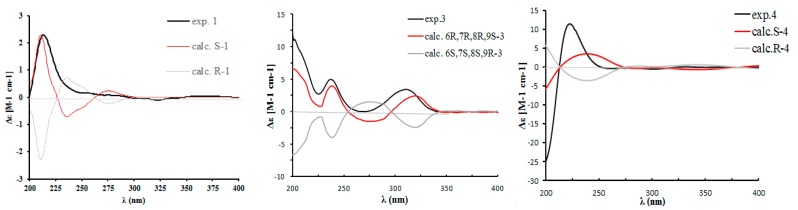
Calculated and experimental electronic circular dichroism (ECD) spectra of 1, 3, and 4 in methanol.

In the ROESY spectrum, correlations from H-10, H-9a, and H_3_-15 to H_3_-16 were observed (Figure 3), which allowed H-10 to be placed on the same side of H_3_-15 and H_3_-16. In order to establish the absolute configuration of compound **1**, density functional theory (DFT) calculations at the APFD/6-311+g (2d, p) level of the ECD spectra were carried out and compared with the experimental ones (Figure 4); their identical spectral profiles supported the *S* configuration of C-10. 

Compound **2** was obtained as a colorless powder with the molecular formula of C_17_H_22_O_3_ according to its negative ion HRESIMS peak at *m*/*z* 273.1487 [M − H]^−^ (calcd 273.1496), indicative of 7 degrees of unsaturation and 14 more mass units with respect to **1**. The ^1^H- and ^13^C-NMR spectroscopic data of compound **2** were quite similar to those of compound **1**, except for an additional methoxy group [*δ*_H_ 3.83 (3H, s); *δ*_C_ 56.1]. In the HMBC spectrum, the methoxy signals had correlations with C-4 (*δ*_C_ 151.4), indicating its connection to C-4 (Figure 2). Finally, the entire structure of compound **2** was elucidated as 4-methylated clavipol A. The absolute configuration of **2** was determined to be identical with that of **1** by comparing their ECD spectra (supplementary Figures S7 and S14), and the compound was given the name clavipol B.

**Figure 5:**
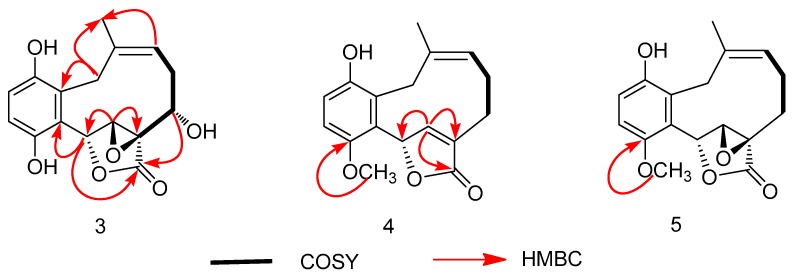
Key ^1^H-^1^H COSY and HMBC correlations for compounds **3**–**5**.

**Figure 6:**
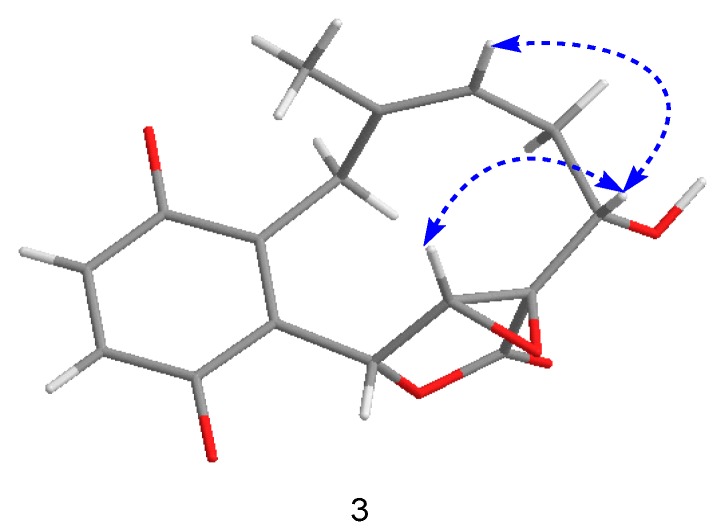
Key nuclear overhauser effect (NOE) correlations for compound **3**.

Compound **3** was isolated as a yellow powder, having the formula of C_16_H_16_O_6_ with nine degrees of unsaturation based on the HRESIMS at *m*/*z* 303.0885 [M − H]^−^ (cal. 303.0874). Overall consideration of 1D-NMR data (Table 2) suggested that compound **3** was a meroterpenoid similar to clavilactone A [13]. In detail, two aromatic protons at *δ*_H_ 6.77(1H, d, *J* = 8.4 Hz, H-2), 6.66(1H, d, *J* = 8.4 Hz, H-3), together with six olefinic carbons at *δ*_C_ 151.0 (C-1), 118.9 (C-2), 115.7 (C-3), 151.0 (C-4), 120.8 (C-5), 128.1 (C-14), indicated the presence of one four-substituted benzene ring. ^13^C-APT NMR signals of *δ*_C_ 77.1 (C-6), 64.7 (C-7), 62.8 (C-8), and 172.8 (C-16) indicated the presence of an α,β-epoxy ɤ-lactone moiety, which was further confirmed by HMBC correlations from H-7(4.02, s) to C-5, C-6, C-8, and C-16 (Figure 5). The methine signal at *δ*_H_ 3.47(1H, dd, *J* = 3.0, 12.0 Hz) and *δ*_C_ 71.3(C-3) indicated that the hydroxyl group was substituted at C-9, which was further supported by the HMBC correlations from *δ*_H_ 3.47 to C-8 (62.8), C-10 (33.4), and C-16 (172.8). By analyzing the COSY spectrum, one proton-bearing structure fragment [=CH–CH_2_–CH–] was readily established (indicated by bold bonds in Figure 2). In the HMBC spectrum, the correlations from H-13b to C-11, C-12, C-14, and C-15 and from H-9 to C-8, C-10, and C-16, indicated that compound **3** was a clavilactone homologue containing hydrobenzene fused to a 10-member carbocycle with α,β-epoxy ɤ-lactone. As a result, compound **3** was established as 9-hydroxyl-substituted clavilactone A. In the ^1^H-NMR spectrum, no vicinal coupling between the two adjacent protons H-6 and H-7 suggested these protons form a dihedral angle of approximately 90°, which means that the relative configurations of C-6, C-7, and C-8 are 6*R*, 7*R*, 8*R* or 6*S*, 7*S*, 8*S*. In the NOESY spectrum, the correlations of H-7a/H-9, H-11, H-13, H_3_-15; H-9/H-11, H-7a, and H-10a/H-13b revealed the close proximity of these protons, and the observed correlation of H-9 to H-7a and H-11 (Figure 6) allowed H-9 to be placed on the side H-9b due to the reported correlations between H-9b to H-7a in clavilactone A [13,17]. The positive cotton effects at 237 and 308 nm showed in the ECD spectrum of **3** (supplementary Figure S21) were in good agreement with the calculated CD spectrum of 6*R*, 7*R*, 8*R*, 9*S* configuration for **3**, as shown in Figure 4. Thus, the absolute configuration of **3** was determined to be 6*R*, 7*R*, 8*R*, 9*S*, and the compound was given the name clavilactone G. 

Compound **4** was isolated as a yellow powder. The HRESIMS displayed an [M − H]^−^ ion peak at *m*/*z* 285.1132 (calcd 285.1132), which showed the molecular formula C_17_H_18_O_4_. The IR spectrum suggested the presence of hydroxyl (3370 cm^−1^) and carbonyl groups (1714 cm^−1^). By intensive comparison of the ^1^H and ^13^C-NMR data (Table 2) with those of clavilactone A, significant differences were the absence of two oxygenated carbon signals and the presence of a double bond [*δ*_H_ 6.80 (1H, s); *δ*_C_ 149.7, 129.2] as well as of a methoxy group [*δ*_H_ 3.83(3H, s); *δ*_C_ 53.1] in **4**. In the HMBC spectrum, the correlations from *δ*_H_ 6.80 to C-6, C-8, and C-16 established that the α,β-epoxy-ɤ-lactone moiety in clavilactone A had been cracked and dehydrated to form an α,β-unsaturated-ɤ-lactone unit in **4**. The methoxy group was attached to C-4 on the basis of the HMBC correlation from protons (*δ*_H_ 3.83) to C-4 (*δ*_C_ 153.1). The experimental ECD spectrum of **4** exhibited the positive cotton effect at 222 nm (supplementary Figure S28), in agreement with the calculated CD spectrum of 6*S* configuration for **4** (Figure 4). Thus, the absolute configuration of **4** was determined as *S*, and the compound was given the name clavilactone H. 

Compound **5** was isolated as a yellow amorphous powder. The HRESIMS displayed an [M − H]^−^ ion peak at *m*/*z* 301.1070 [M − H]^−^ (calcd 301.1081), which showed the molecular formula C_17_H_18_O_5_ and 14 more mass units than clavilactone A [13]. Its IR bands at 3436 cm^−1^ and 1749cm^−1^ suggested the existence of hydroxyl and carbonyl groups. Overall consideration of ^1^H and ^13^C data of **5** indicated compound **5** could be a methylation derivative of clavilactone A, which was further supported by its HMBC spectrum. In the HMBC spectrum, the correlation between *δ*_H_ (3.83, s) and C-4 (*δ*_C_ 153.1) established the presence of a methoxy group (*δ*_C_ 57.1) attaching to C-4 in compound **5** (Figure 5). Considering the identical CD spectra of compound **5** and clavilactone A (supplementary Figures S34 and S35), compound **5** should have the same absolute configuration of calvilactone A, and was given the name clavilactone I.

Furthermore, all isolated compounds (**1**–**5**) were evaluated for their cytotoxicity against three human tumor cell lines (Hela, SGC-7901, and SHG-44) in vitro by treatment for 48 h, using the MTT assay [19]; cisplatin was used as the positive control drug. The results of cytotoxicity are displayed in Table 3. Compound **4** exhibited moderate cytotoxic activity against Hela and SGC-7901 tumor cell lines, with IC_50_ values of 23.5 and 14.5 µM, respectively.

## 3. Discussion

The chemical investigation of the fungus *C. clavipes* led to the isolation of five new meroterpenoids, clavipols A–B (**1**–**2**) with a 12-membered ether ring and clavilactones G–I (**3**–**5**) having a 10-membered carbocycle connected to a hydroquinone and an α,β-epoxy/unsaturated lactone. This study contributes to broadening the list of known chemically diverse meroterpenoids from the fungus *C. clavipes*.

Until now, only about 20 naturally occurring meroterpenoids with a benzo-fused 10-membered carbocycle unit have been isolated from plants and fungi [17]. These meroterpenoids have been shown to display potent cytotoxic activities [20]. For example, terreumols A, isolated from the mushroom *Tricholoma terreum*, displayed potent cytotoxic activity against A-549 cancer cell line, with an IC_50_ value of 4.2 μM [20]. Compared with the cytotoxic activities of compounds **3** and **5**, compound **4** exhibited moderate cytotoxic activity against Hela and SGC-7901 cancer cell lines, with IC_50_ values of 23.5 and 14.5 µM, respectively. Therefore, we speculate that different degrees of oxidation of such compounds may affect their cytotoxic activities. 

## 4. Materials and Methods 

### 4.1. General Experimental Procedures

1D and 2D-NMR spectra were obtained with a Bruker AV 600 NMR spectrometer (chemical shift are presented as δ values with TMS as the internal standard) (Bruker, Billerica, Germany). HRESIMS was performed on a Q-tof spectrometer (Waters, Milford, MA, USA). UV and IR data were obtained using a Shimadzu UV2550 spectrophotometer and a FTIR-8400S spectrometer (Shimadzu, Kyoto, Japan), respectively. CD spectra were obtained using a JASCO J-815 spectropolarimeter (Tokyo, Japan). Thin-layer chromatography (TLC) was performed on pre-coated silica gel GF254 (Zhi Fu Huang Wu Pilot Plant of Silica Gel Development, Yantai, China). Semi-preparative HPLC was conducted on an analytic LC equipped with a pump of P230 and a DAD detector of 230+ (Ellte, Dalian, China) with a C_18_ ODS-A (5 µm, YMC, Kyoto, Japan). Column chromatography used silica gel columns (200–300 mesh, Qingdao Marine Chemical plant, Qingdao, China). All solvents used were of analytical grade (Beijing Chemical Plant, China).

### 4.2. Computational Methods

The ECD calculations were carried out using Gaussian 09 program (Inc., WALLINGFORD, CT, USA). Conformers were generated by MMFF94s force field, each conformer was optimized with the HF/6-31G(d) method, and further optimized with the DFT method at the B3LYP/6-311+g(d, p) level. Frequency calculations were also performed at the same level to confirm that each optimized conformer was true minimum and to estimate their relative thermal free energy (ΔG) at 298.15 K. Conformers with the Boltzmann distribution over 1% were chosen for ECD calculations in methanol at the APFD/6-311+g(2d, p) level. The ECD spectra were simulated by the SpecDis program. To obtain the final conformationally averaged data, the simulated spectra of the predominant conformers were averaged according to the Boltzmann distribution theory. 

### 4.3. Fungal Material 

The fruiting bodies of *Clitocybe clavipes* were collected from Hotan Prefecture, Xinjiang Uygur Autonomous Region, China, in July 2018. The fungus was identified by Prof. Leiling Shi, Xinjiang Institute of Chinese and Ethnic Medicine, where a voucher specimen of *C. clavipes* (No. 201812) was preserved. 

### 4.4. Extraction and Isolation

The dried fruiting bodies of *C. clavipes* (0.6 kg) were macerated three times with EtoAc. The solvents were filtrated and evaporated in vacuum to give the total extract (45 g), and this residue was subjected to silica-gel (200–300 mesh) column chromatography (CC) with two gradient systems (ether/EtOAc 30:1, 10:1, 5:1,1:1; CH_2_Cl_2_/MeOH 20:1, 10:1, 5:1, 1:1, *v*/*v*) to give 8 fractions (F1–F8). F3 was purified by semi-preparative HPLC (CH_3_CN/H_2_O 60:49, *v*/*v*) to yield **1** (3 mg, *t*_R_ = 15.5 min) and **2** (2 mg, *t*_R_ = 17.1 min). F4–5 were subjected to C-18 reversed-phase (RP) silica-gel CC using MeOH/H_2_O in a linear gradient (30:70, 45:55, 60:40, 80:20, 100:0, *v*/*v*) to obtain 5 fractions (F4–5.a–F4–5.e). F4–5.d was purified by semi-preparative HPLC with CH_3_CN-H_2_O as mobile phase (45:55, *v*/*v*), to give **3** (2 mg, *t*_R_ = 12.4 min), **4** (2 mg, *t*_R_ = 20.0 min), and **5** (3 mg, *t*_R_ = 22.1 min). 

The structures of compounds **1**–**5** were determined by HRESIMS, UV, IR, 1D and 2D-NMR spectra.

Clavipol A (**1**), colorless powder; [α]^25^_D_ +18.6 (c 0.11, MeOH); UV(MeOH) λ_max_ (log ε) 204(3.54), 291(3.2) nm; IR(KBr) v_max_ 3417, 2917, 2851,1596, 1385, 1118, 768, 544 cm^−1^; ^1^H and ^13^C-NMR data see Table 1; (-)HRESIMS *m*/*z* 259.1329 [M − H]^−^ (calcd for 259.1340).

Clavipol B (**2**), colorless powder; [α]^25^_D_ +18.0 (c 0.10, MeOH); UV(MeOH) λ_max_ (log ε) 203(3.3), 290(3.1) nm; IR(KBr) v_max_ 3418, 2914, 2849,1593, 1383, 1115, 763, 546 cm^−1^; ^1^H and ^13^C NMR data see Table 1; (-)HRESIMS *m*/*z* 273.1487 [M − H]^−^ (calcd 273.1496).

Clavilactone G (**3**), yellow powder; [α]^25^_D_ +50.8 (c 0.10, MeOH); UV(MeOH) λ_max_ (log ε) 235(3.91), 310(3.52) nm; IR(KBr) v_max_ 3440, 2944, 1776, 1487, 1419, 1233, 1210, 802 cm^−1^; ^1^H and ^13^C-NMR data see Table 2; (-)HRESIMS *m*/*z* 303.0885 [M − H]^−^ (cal. 303.0874).

Clavilactone H (**4**), yellow powder; [α]^25^_D_ −21.8 (c 0.06, MeOH); UV(MeOH) λmax (log ε) 209(3.91), 281(3.52) nm; IR(KBr) v_max_ 3370, 2925, 2853, 1743, 1599, 1453, 1267, 1076, 807cm^−1^; ^1^H and ^13^C NMR data see Table 2; (+)HRESIMS *m*/*z* 285.1132(calcd 285.1132).

Clavilactone I (**5**), yellow powder; [α]^25^_D_ +90.5 (c 0.12, MeOH); UV(MeOH) λmax (log ε) 236(3.70), 310(3.56) nm; IR(KBr) v_max_ 3435, 2930, 1749, 1487, 1260, 1152, 1021, 800 cm^−1^; ^1^H and ^13^C NMR data see Table 2; (-)HRESIMS *m*/*z* 301.1070 [M − H]^−^ (calcd 301.1081).

### 4.5. Cytotoxicity Assays

Compounds **1**–**5** were evaluated for their cytotoxic activity by the MTT method using Hela, SGC-7901, and SHG-44 cancer cell lines. Cells were grown in DMEM medium and cultured at a density of 6 × 10^4^ cells/mL per well in a 96-well microtiter plate. Then, different concentrations of the isolated compounds dissolved in dimethyl sulfoxide (DMSO) were added to each well. Each concentration was tested in triplicate. After incubation at 37 °C in 5% CO_2_ for 48 h, 10 μL of MTT was added to each well, and incubation was continued for additional 4 h. Then, the liquid was removed from the wells and DMSO was added to each well. The absorbance was recorded on a microplate reader at a wavelength of 570 nm.

## Figures and Tables

**Table 1:** NMR spectral data of **1** and **2** (600 MHz for ^1^H-NMR and 150 MHz for ^13^C-NMR).

	1		2	
No.	*δ*_H,_ (*J* in Hz)	*δ*_C_	*δ*_H,_ (*J* in Hz)	*δ*_C_
1	-	151.6	-	151.6
2	6.56, dd (3.0, 8.4)	115.3	6.64, dd (3.0, 8.4)	114.7
3	6.60,d (8.4)	115.8	6.69, d (8.4)	111.3
4	-	147.1	-	151.4
5	-	127.4	-	129.9
6	3.04, dd (8.4, 16.8),b 3.34, dd (7.2, 16.8),a	25.7	3.03, dd (8.4, 16.8),b 3.39, dd (7.2, 16.8),a	25.9
7	5.74, t (7.2)	126.6	5.73, t (7.2)	127.2
8	-	135.7	-	135.3
9	2.36, t (12),b 2.71, dd (4.2, 12),a	48.6	2.35, t (12),b 2.71, dd (4.2, 11.4),a	48.7
10	4.68, m	66.0	4.68, m	66.0
11	5.56, d (10.2)	135.4	5.57, d (10.2)	135.3
12	-	136.5	-	136.6
13	4.46, d (12.6), b 4.64, d (12.6), a	76.2	4.47, d (12.6), b 4.66, d (12.6), a	76.2
14	7.23, d (3.0)	117.8	7.30, d (3.0)	118.0
15	1.56, s	13.4	1.56, s	13.4
16	1.24, s	16.1	1.23, s	16.1
-OCH_3_	-	-	3.78, s	56.1

**Table 2:** NMR spectral data of **3**–**5** (600 MHz for ^1^H-NMR and 150 MHz for ^13^C-NMR).

	3 ^a^		4 ^b^		5 ^b^	
No.	*δ*_H,_ (*J* in Hz)	*δ*_C_	*δ*_H,_ (*J* in Hz)	*δ*_C_	*δ*_H,_ (*J* in Hz)	*δ*_C_
1	-	151.0	-	149.1	-	148.9
2	6.77, d (8.4)	118.9	6.79, d (8.4)	117.2	6.83, d (9.0)	117.5
3	6.66, d (8.4)	115.7	6.75, d (8.4)	111.1	6.75, d (9.0)	110.8
4	-	151.0	-	153.1	-	153.1
5	-	120.8	-	120.5	-	122.4
6	6.30, s	77.1	6.84, s	76.1	6.37, s	74.6
7	4.02, s	64.7	6.80, s	149.7	4.01, s	63.8
8	-	62.8	-	129.2	-	61.8
9	3.47,dd (3.0,12.0), m	71.3	1.87, m, b 2.74, m, a	25.6	1.27, m, b 2.73, m, a	25.3
10	2.27, m, b 2.85, m, a	33.4	1.97, m, b 2.23, m, a	24.9	2.18, m, b 2.48, m, a	22.7
11	5.27, t (7.8)	119.8	5.12, t (7.8)	121.4	5.27, t (8.4)	122.4
12	-	141.7	-	140.1	-	137.8
13	3.02, d (15.6), b 3.69,d (15.6), a	28.5	2.58, d (15.0), b 3.32, d (15.0), a	27.0	3.09, d (15.6), b 3.693,d (15.6), a	27.9
14	-	128.1	-	128.0	-	128.4
15	1.56, s	22.1	1.59, s	22.1	1.56, s	21.6
16	-	172.8	-	175.2	-	172.8
-OCH_3_	-	-	3.83, s	57.1	3.80, s	56.8

^a^ Spectral data were recorded in CD_3_OD; ^b^ Spectral data were recorded in CDCl_3_.

**Table 3:** In vitro cytotoxic activity of compounds **1**–**5**.

Compounds	IC_50_ (μM)		
	Hela	SGC-7901	SHG-44
1	63.2 ± 0.43 ^a^	44.1± 0.5	>100
2	38.6 ± 1.3	51.2 ± 0.7	>100
3	>100	62.2 ± 0.4	>100
4	23.5 ± 0.4	14.5 ± 1.2	53.9 ± 2.4
5	>100	84.2 ± 3.1	>100
Cisplatin	2.7 ± 0.04	1.1 ± 0.05	1.8 ± 0.03

^a^ The values presented are the means ± SD of triplicate experiments.

## Supplementary Materials

Supplementary Materials are available online.

## Abbreviations

The following abbreviations are used in this manuscript:

IR	Infrared
NMR	Nuclear magnetic resonance
HR-ESI-MS	High resolution electrospray ionization mass spectroscopy
HMBC	Heteronuclear multiple bond correlation
HSQC	Heteronuclear single quantum correlation
COSY	Homonuclear chemical shift Correlation Spectroscopy
NOESY	Nuclear overhauser effect spectroscopy
ROESY	Rotating frame overhauser effect spectroscopy
APFD	Austin-Frisch-Petersson functional with dispersion
TMS	Tetramethylsilane
ODS	Octadecyl silane
HPLC	High performance liquid chromatography
CH_2_Cl_2_	Dichloromethane
EtOAc	Ethyl acetate
MeOH	Methanol
MTT	3-(4,5-Dimethylthiazol-2-yl)-2,5-diphenyltetrazolium bromide
