# Supplementary material for: Five New Meroterpenoids from the Fruiting Bodies of the Basidiomycete Clitocybe clavipes with Cytotoxic Activity
Source: Molecules. 2019 Nov 6;24(22):4015. doi: 10.3390/molecules24224015 (PMC6891274; doi:10.3390/molecules24224015)
Supplement: Supplementary file 1 [file molecules-24-04015-s001.pdf]

Five New Meroterpenoids from the Fruiting Bodies of the Basidiomycete *Clitocybe clavipes* with Cytotoxic Activity

Zhaocui Sun [1], Xudong Xu [1], Hanqiao Liang [2], Xinyi Xia [1], Guoxu Ma [1,*] and Leiling Shi [3,*]

[1] Key Laboratory of Bioactive Substances and Resource Utilization of Chinese Herbal Medicine, Ministry of Education, Institute of Medicinal Plant Development, Peking Union Medical College and Chinese Academy of Medical Sciences, Beijing 100193, China; flydancingsun@163.com (Z.S.); xdxu2012@163.com (X.d.X.); xiaxinyi163@163.com (X.y.X.)

[2] Department of Biomedicine, Beijing City University, Beijing 100094; yaoxue2016@yeah.net

[3] Xinjiang Institute of Chinese and Ethnic Medicine, Urumqi 830002, China

* Correspondence: mgxfl8785@163.com (G.-X.M.); shileiling@sina.com(L.-L.S.)

Abstract: Five new meroterpenoids, clavipols A–B (**1**–**2**) with a 12-membered ether ring and clavilactones G–I (**3**–**5**) having a 10-membered carbocycle connected to a hydroquinone and an α,β-epoxy/unsaturated lactone, were obtained from the fruiting bodies of the basidiomycete *Clitocybe clavipes*. Their structures were determined by comprehensive analysis of their spectroscopic data, and the absolute configuration of **1** was established by quantum chemical calculations of electronic circular dichroism (ECD). All the isolated compounds (**1**–**5**) were tested for their cytotoxic activity against three human tumor cell lines (Hela, SGC-7901, and SHG-44) in vitro after treatment for 48 h. Compound **4** exhibited moderate cytotoxic activity against Hela and SGC-7901 tumor cell lines, with IC_{50} values of 23.5 and 14.5 μM, respectively.

Keywords: meroterpenoids; *Clitocybe clavipes*; basidiomycete; cytotoxicity

List of Figures S1-S34

Figure S1. ^{1}H-NMR (600 MHz, $CDCl_3$) spectrum of the new compound **1**

Figure S2. ^{13}C-APT (150 MHz, $CDCl_3$) spectrum of the new compound **1**

Figure S3. ^{1}H-^{1}H COSY spectrum of the new compound **1**

Figure S4. HSQC spectrum of the new compound **1**

Figure S5. HMBC spectrum of the new compound **1**

Figure S6. ROESY spectrum of the new compound **1**

Figure S7. CD spectrum of the new compound **1**

Figure S8. ^{1}H-NMR (600 MHz, $CDCl_3$) spectrum of the new compound **2**

Figure S9. ^{13}C-APT (150 MHz, $CDCl_3$) spectrum of the new compound **2**

Figure S10. ^{1}H-^{1}H COSY spectrum of the new compound **2**

Figure S11. HSQC spectrum of the new compound **2**

Figure S12. HMBC spectrum of the new compound **2**

Figure S13. ROESY spectrum of the new compound **2**

Figure S14. CD spectrum of the new compound **2**

Figure S15. ^{1}H-NMR (600 MHz,CD_3OD) spectrum of the new compound **3**

Figure S16. ^{13}C-APT (150 MHz, CD_3OD) spectrum of the new compound **3**

Figure S17. ^{1}H-^{1}H COSY spectrum of the new compound **3**

Figure S18. HSQC spectrum of the new compound **3**

Figure S19. HMBC spectrum of the new compound **3**

Figure S20. NOESY spectrum of the new compound **3**

Figure S21. CD spectrum of the new compound **3**

Figure S22. ^{1}H-NMR (600 MHz, $CDCl_3$) spectrum of the new compound **4**

Figure S23. ^{13}C-APT (150 MHz, $CDCl_3$) spectrum of the new compound **4**

Figure S24. ^{1}H-^{1}H COSY spectrum of the new compound **4**

Figure S25. HSQC spectrum of the new compound **4**

Figure S26. HMBC spectrum of the new compound **4**

Figure S27. ROESY spectrum of the new compound **4**

Figure S28. CD spectrum of the new compound **4**

Figure S29. ^{1}H-NMR (600 MHz, $CDCl_3$) spectrum of the new compound **5**

Figure S30. ^{13}C-APT (150 MHz, $CDCl_3$) spectrum of the new compound **5**

Figure S31. ^{1}H-^{1}H COSY spectrum of the new compound **5**

Figure S32. HSQC spectrum of the new compound **5**

Figure S33. HMBC spectrum of the new compound **5**

Figure S34. CD spectrum of the new compound **5**

Figure S35. CD spectrum of clavilactone A

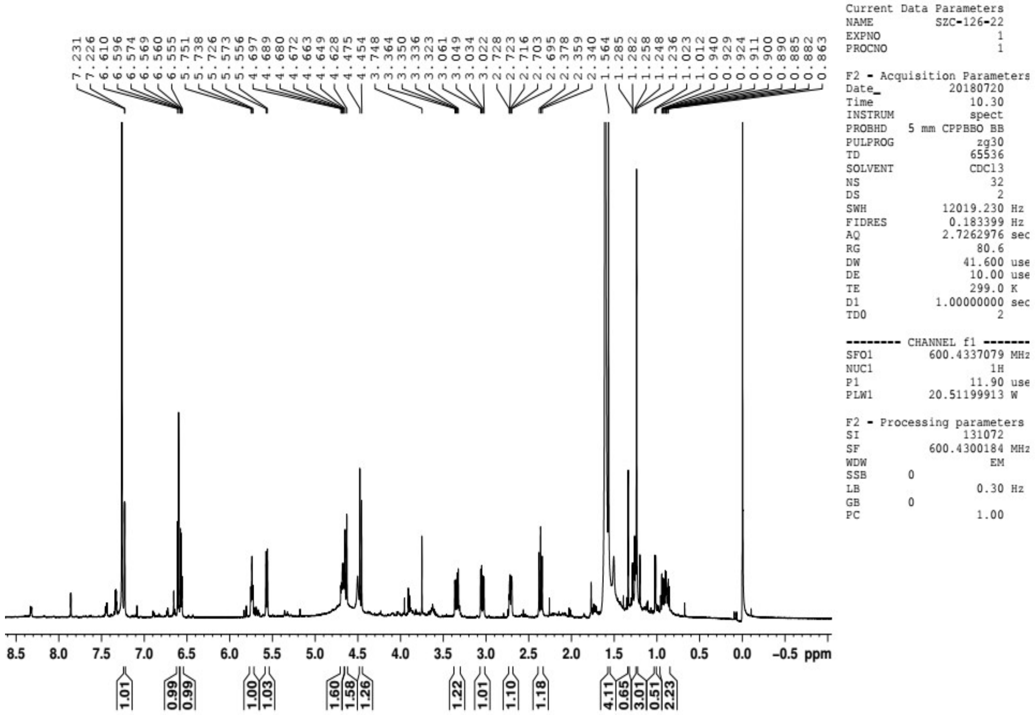

Figure S1. ^{1}H-NMR (600 MHz, $CDCl_3$) spectrum of the new compound **1**

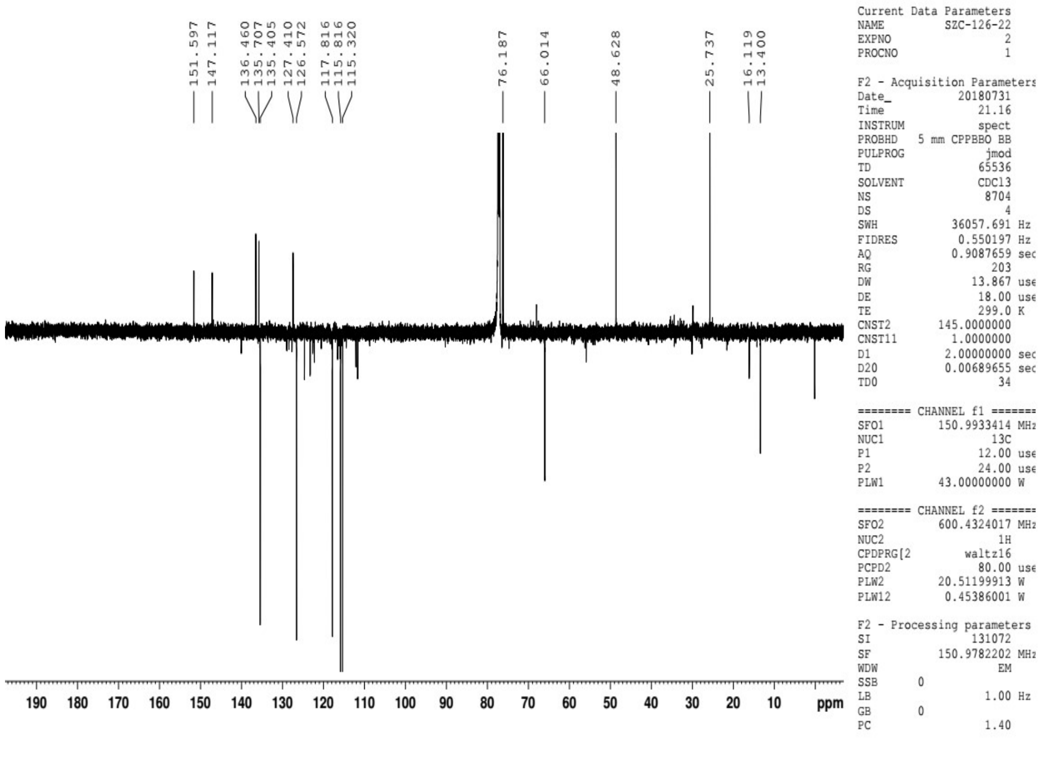

Figure S2. ^{13}C-APT (150 MHz, $CDCl_3$) spectrum of the new compound **1**

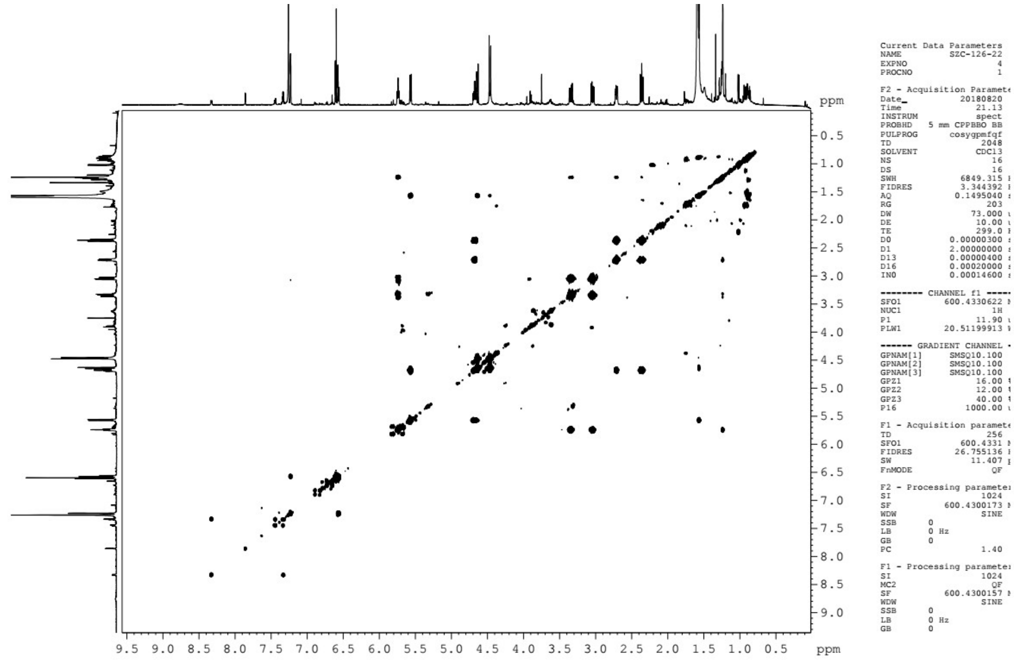

Figure S3. ^{1}H-^{1}H COSY spectrum of the new compound **1**

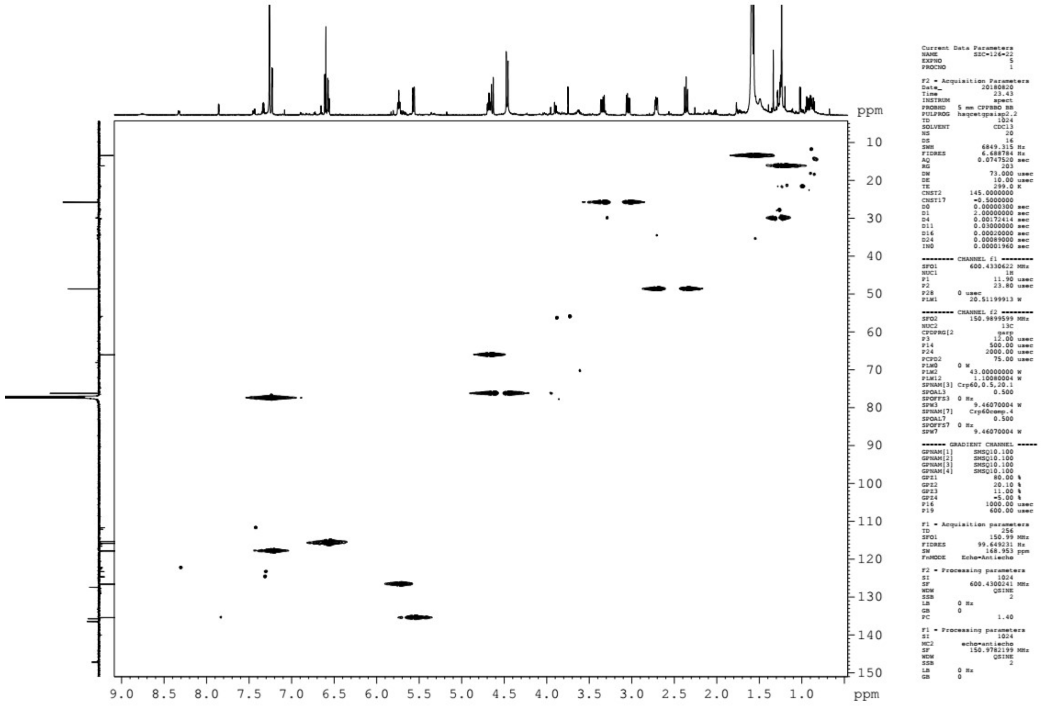

Figure S4. HSQC spectrum of the new compound **1**

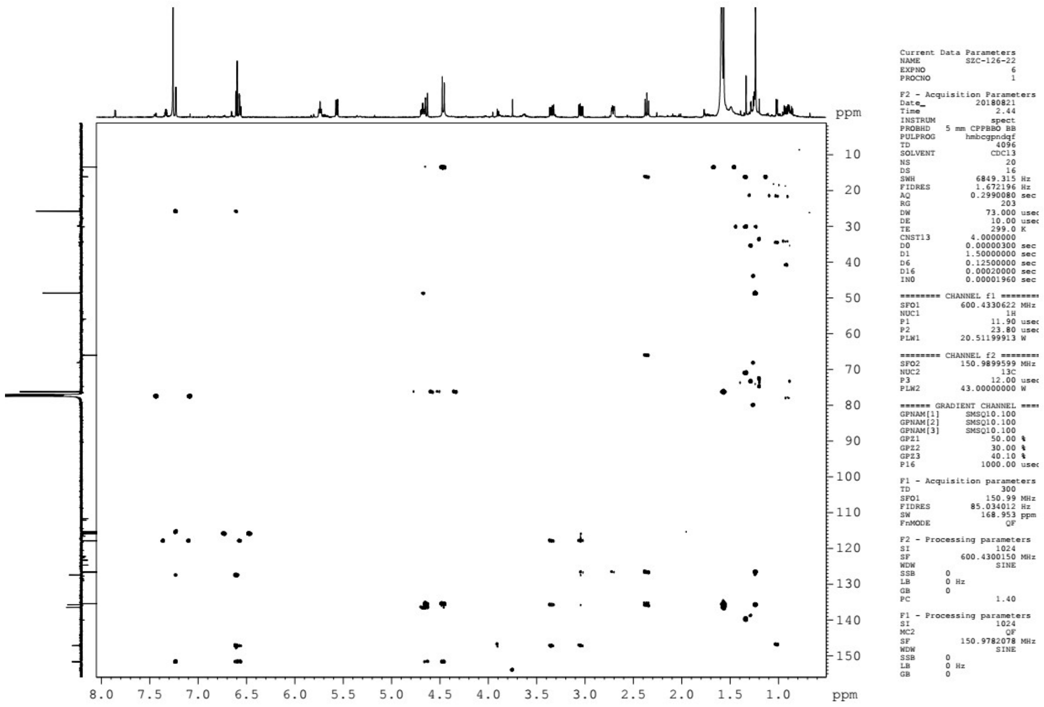

Figure S5. HMBC spectrum of the new compound **1**

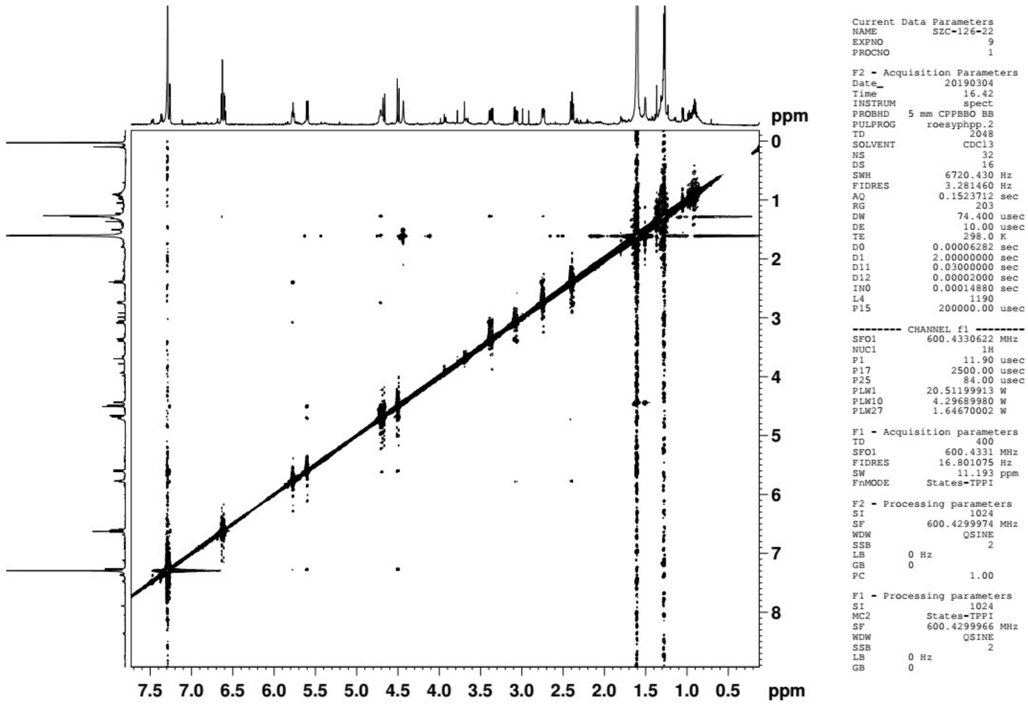

Figure S6. ROESY spectrum of the new compound **1**

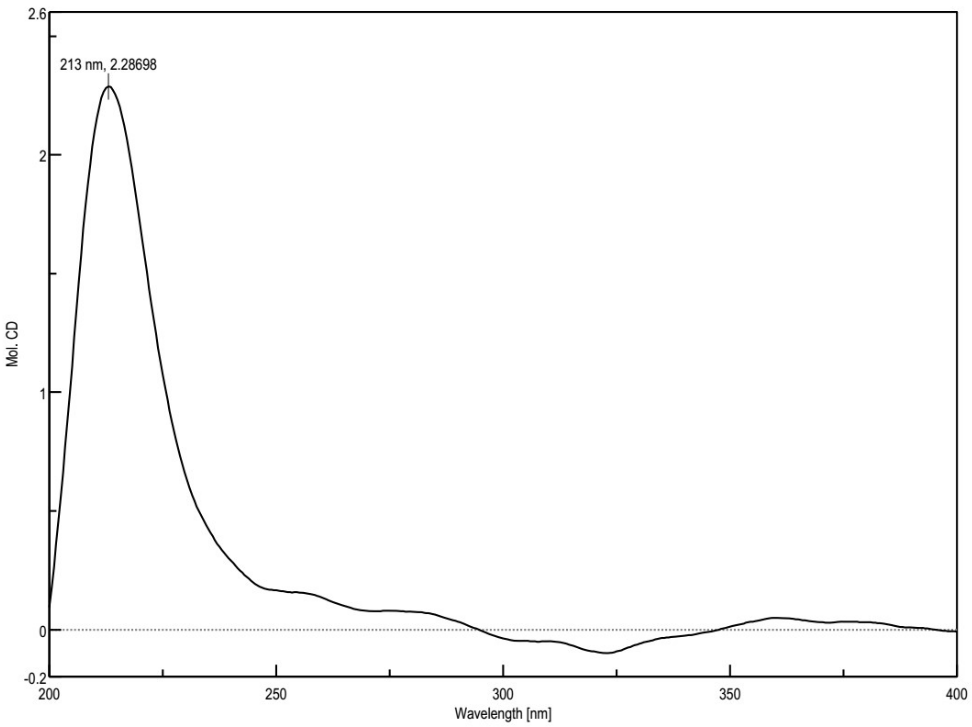

Figure S7. CD spectrum of the new compound **1**

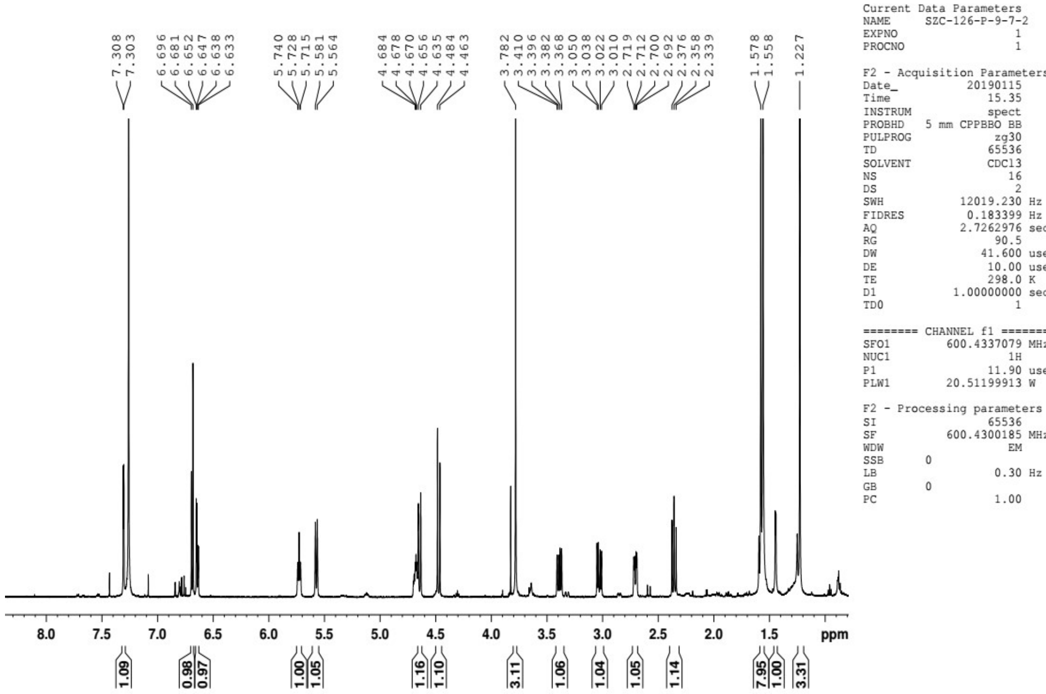

Figure S8. ^{1}H-NMR (600 MHz, $CDCl_3$) spectrum of the new compound **2**

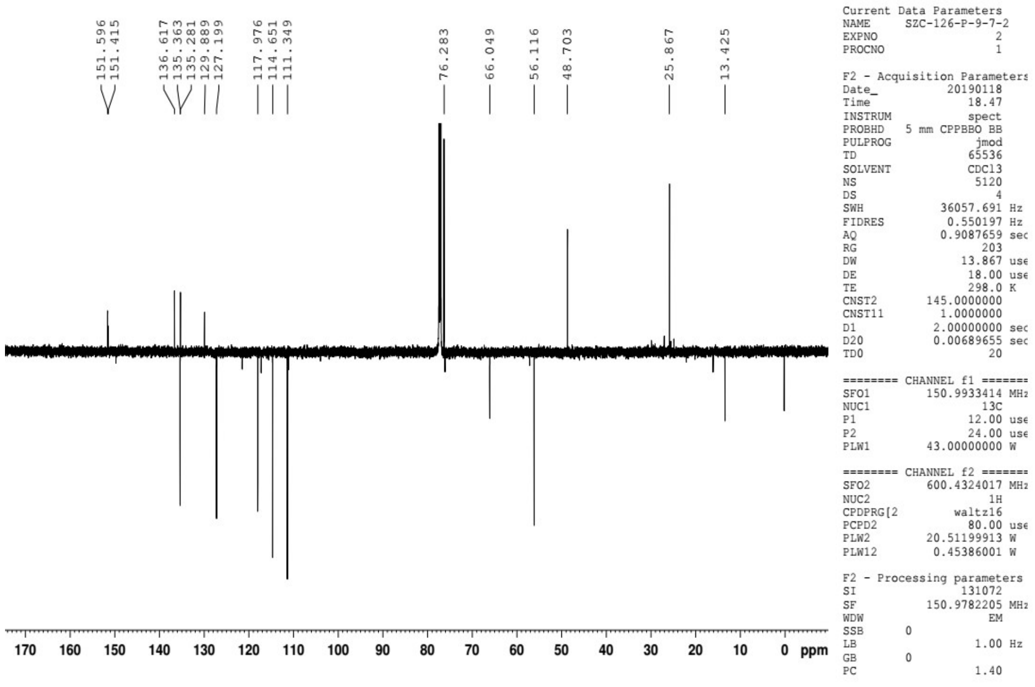

Figure S9. ^{13}C-APT (150 MHz, $CDCl_3$) spectrum of the new compound **2**

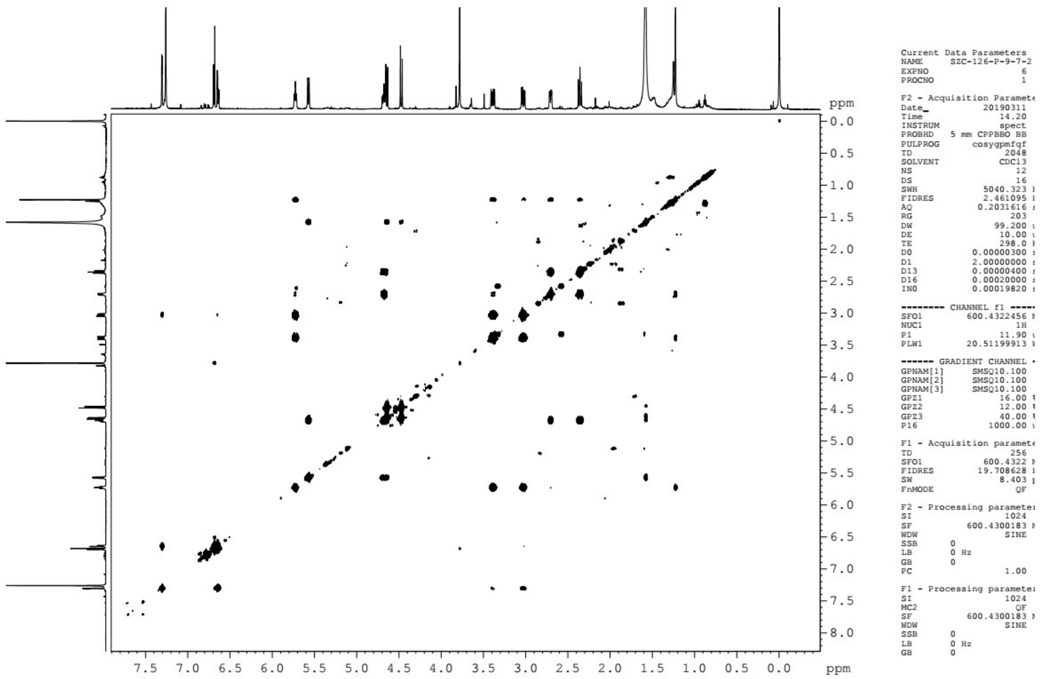

Figure S10. ^{1}H-^{1}H COSY spectrum of the new compound **2**

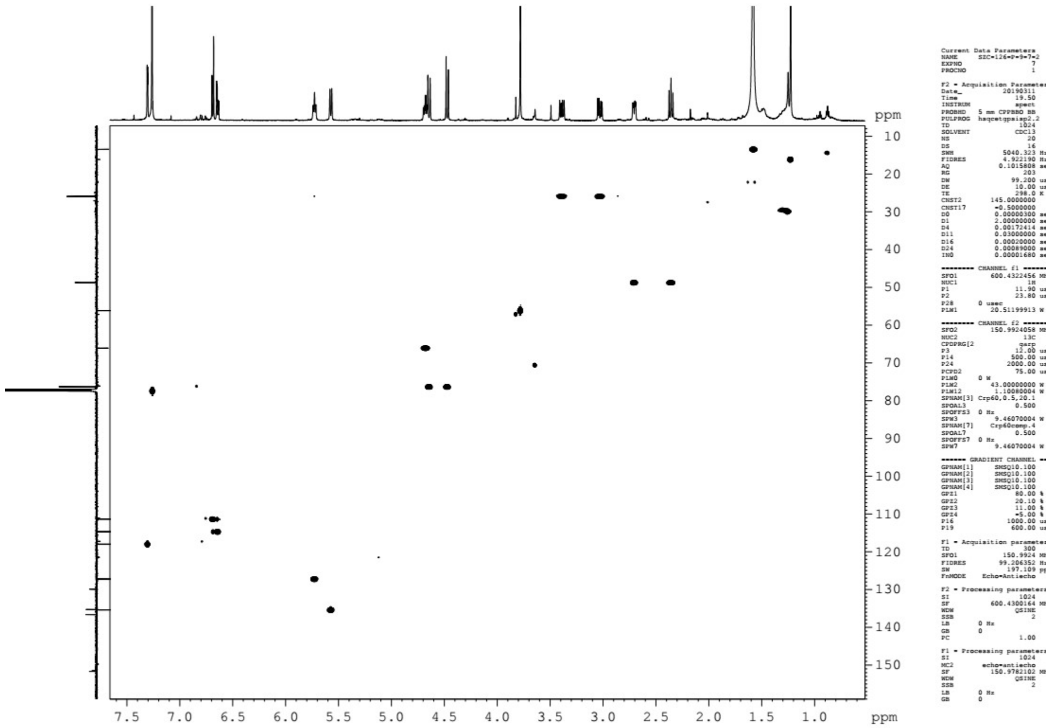

Figure S11. HSQC spectrum of the new compound **2**

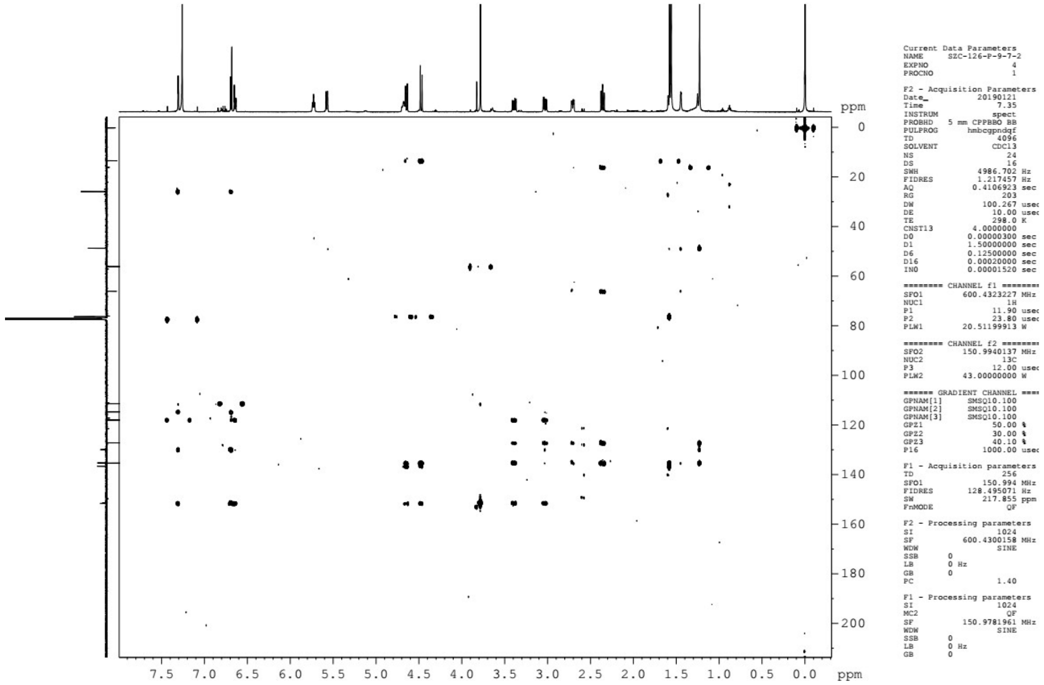

Figure S12. HMBC spectrum of the new compound **2**

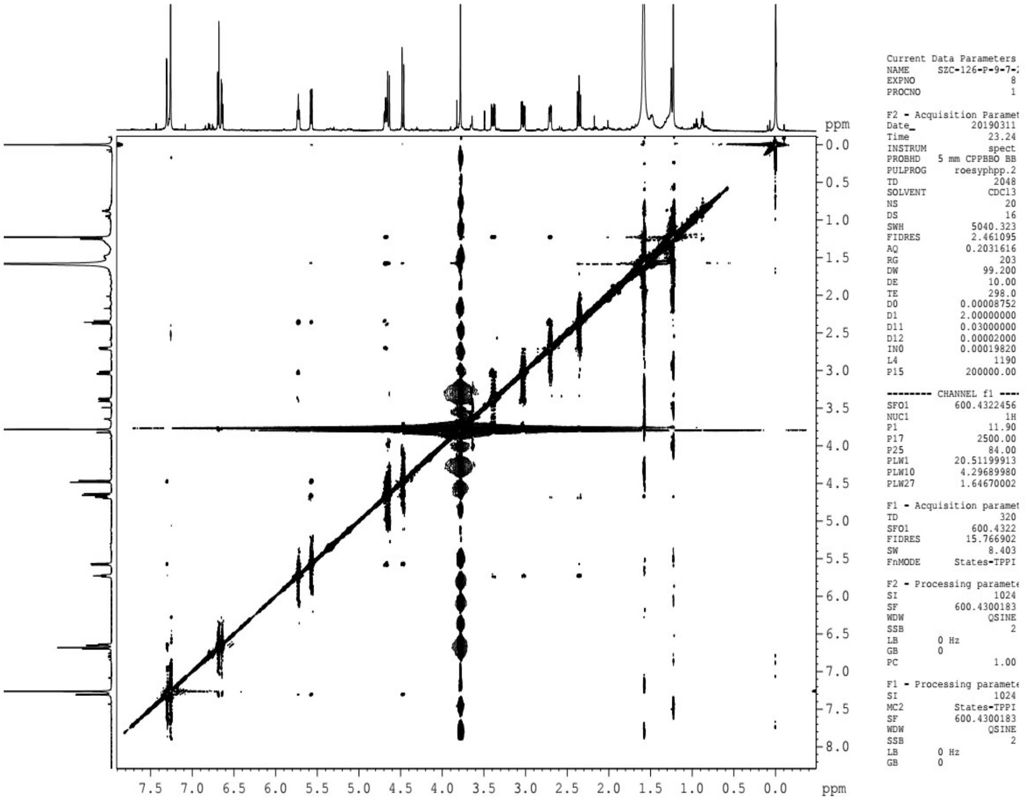

Figure S13. ROESY spectrum of the new compound **2**

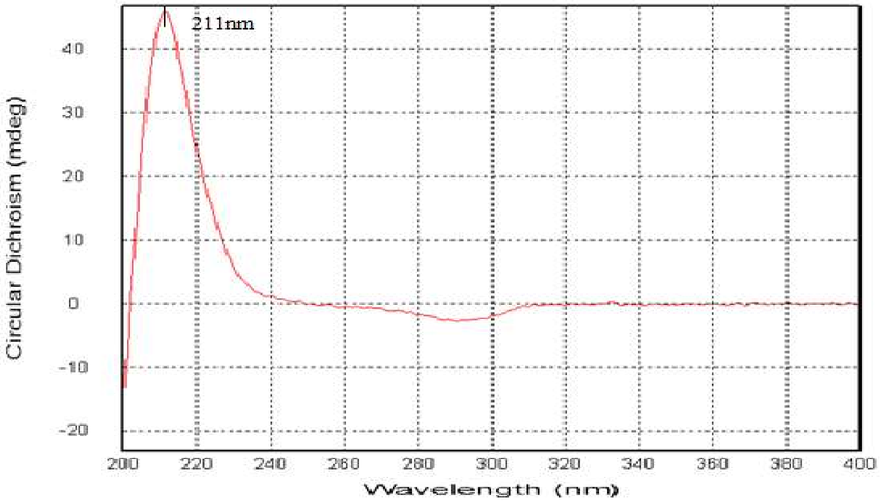

Figure S14. CD spectrum of the new compound **2**

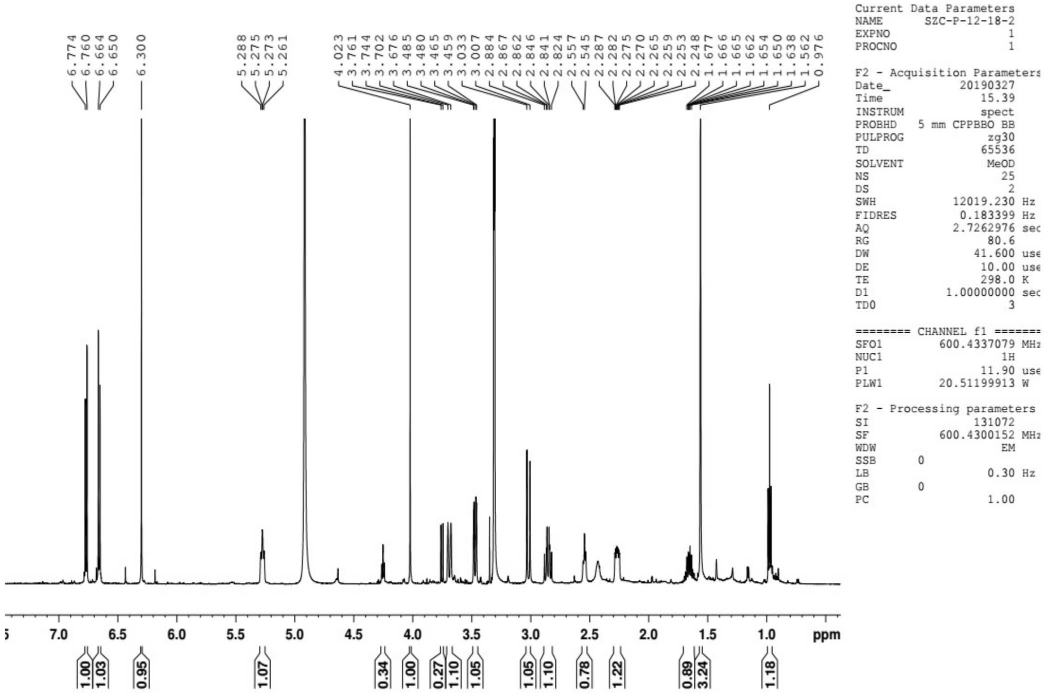

Figure S15. ^{1}H-NMR (600 MHz, CD_3OD) spectrum of the new compound **3**

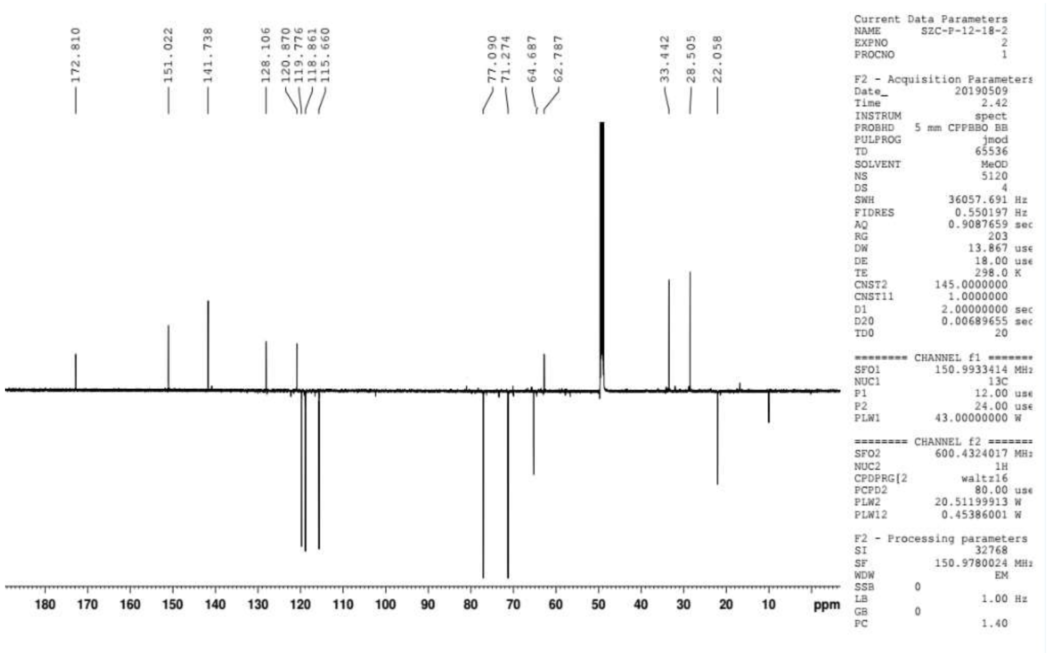

Figure S16. ^{13}C-APT (150 MHz, CD_3OD) spectrum of the new compound **3**

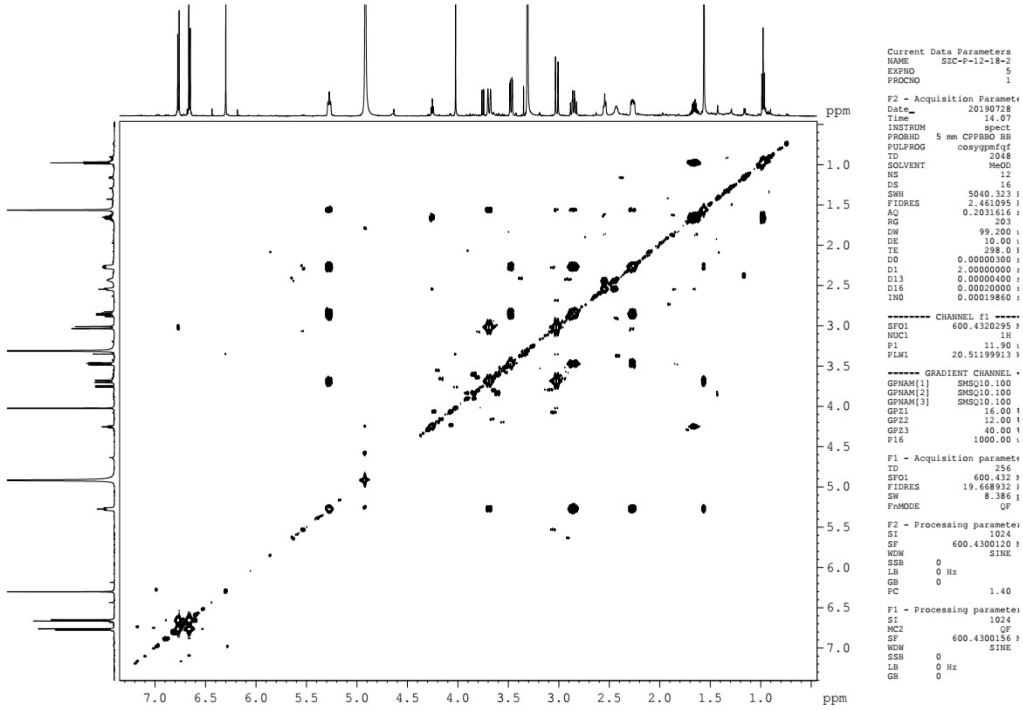

Figure S17. ^{1}H-^{1}H COSY spectrum of the new compound **3**

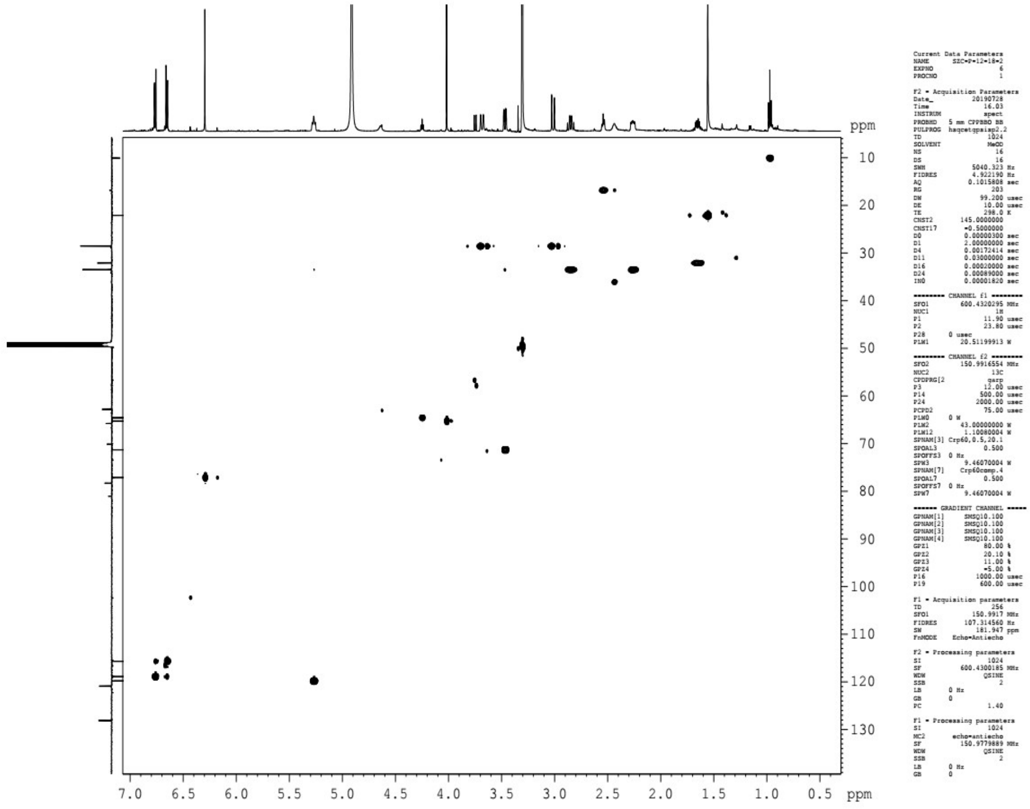

Figure S18. HSQC spectrum of the new compound **3**

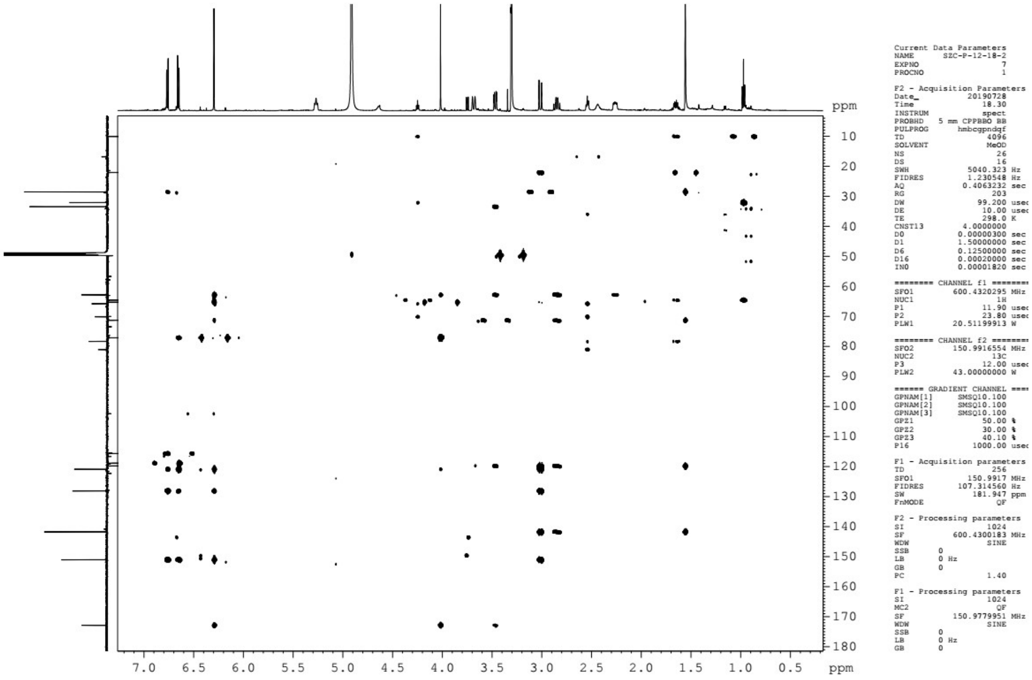

Figure S19. HMBC spectrum of the new compound **3**

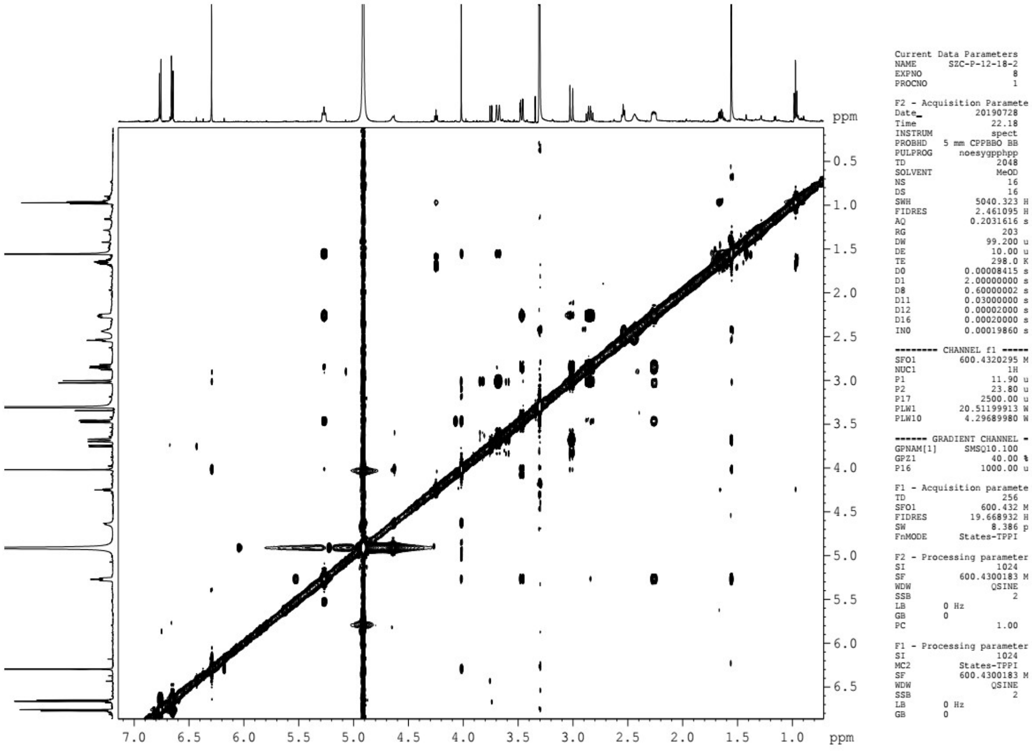

Figure S20. NOESY spectrum of the new compound **3**

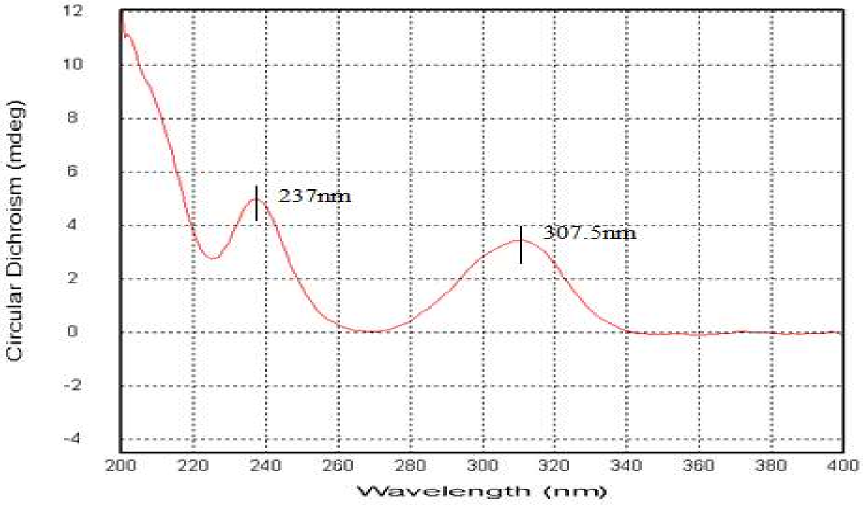

Figure S21. CD spectrum of the new compound **3**

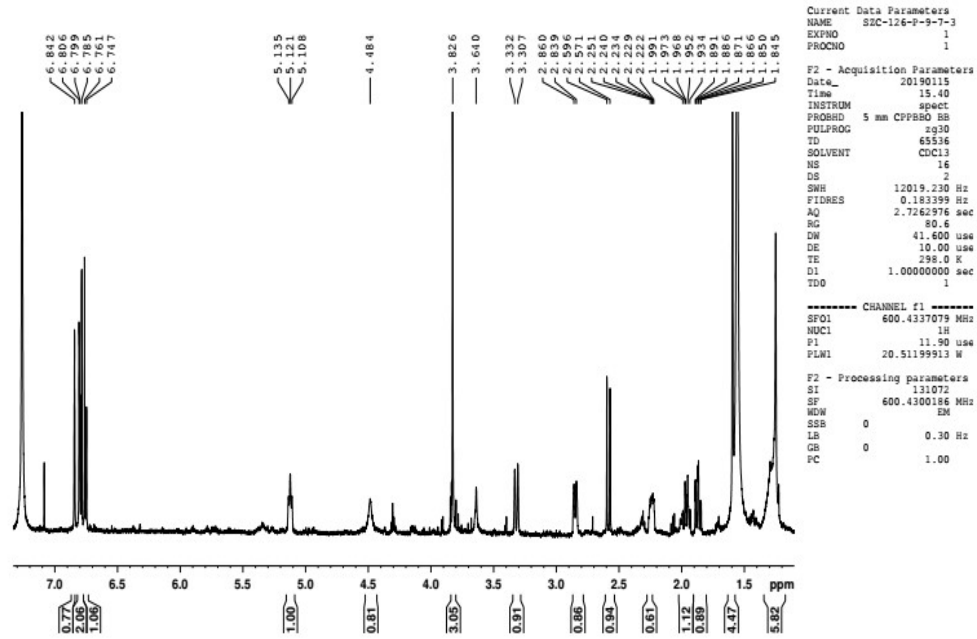

Figure S22. ^{1}H-NMR (600 MHz, $CDCl_3$) spectrum of the new compound **4**

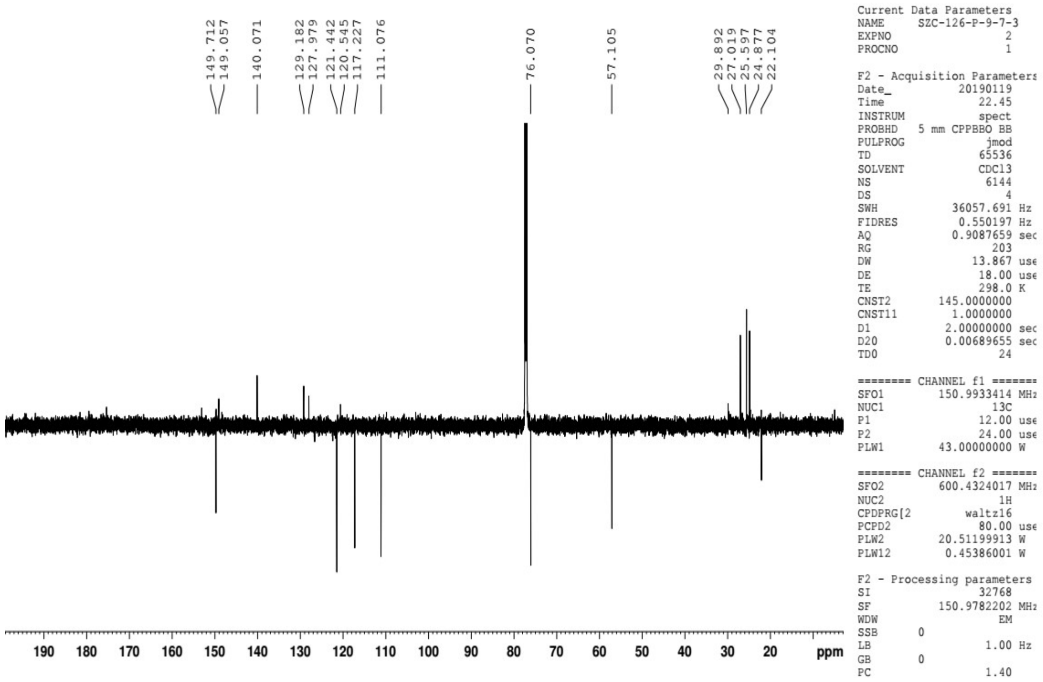

Figure S23. ^{13}C-APT (150 MHz, $CDCl_3$) spectrum of the new compound **4**

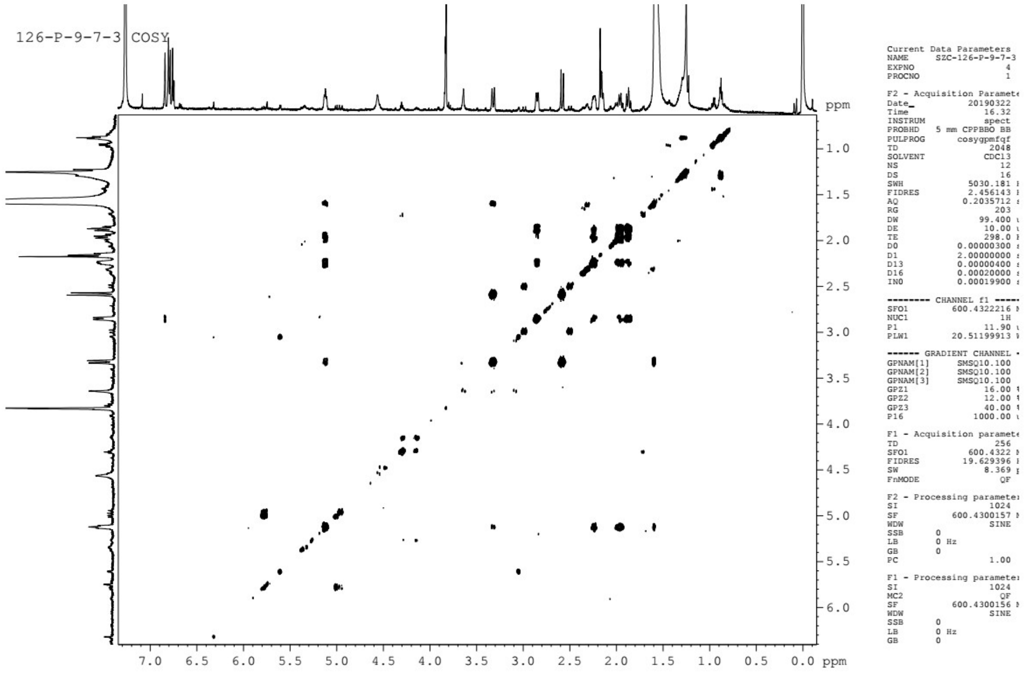

Figure S24. ^{1}H-^{1}H COSY spectrum of the new compound **4**

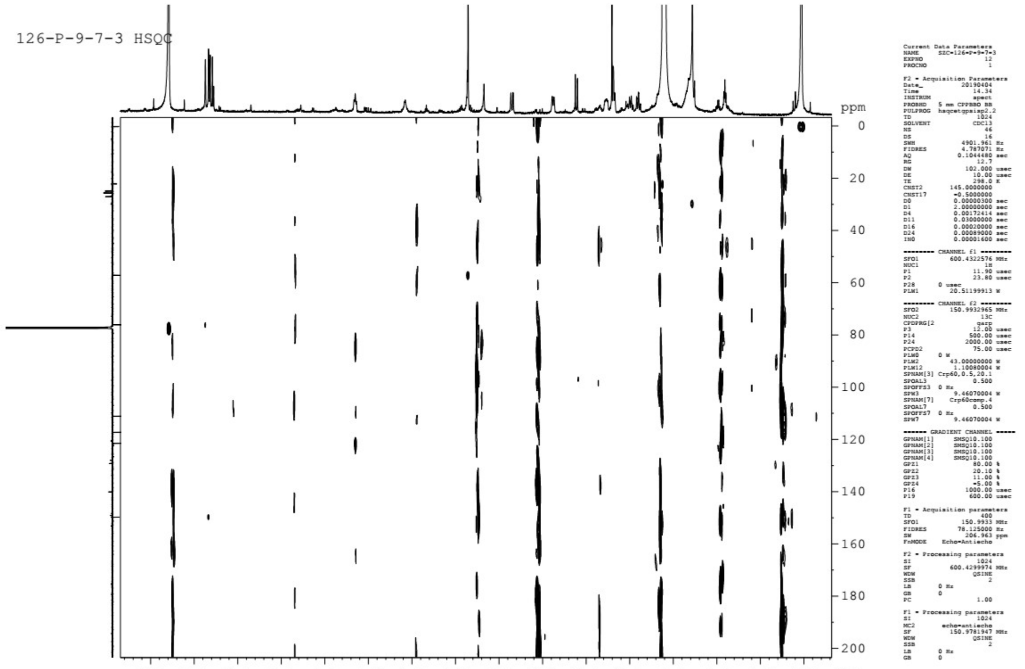

Figure S25. HSQC spectrum of the new compound **4**

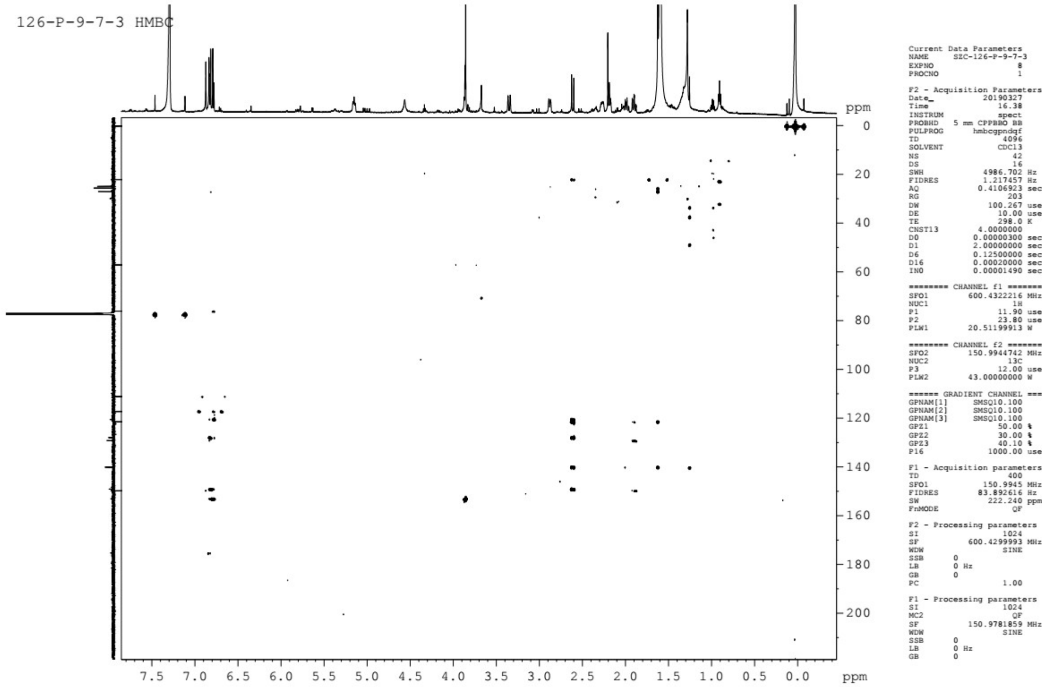

Figure S26. HMBC spectrum of the new compound **4**

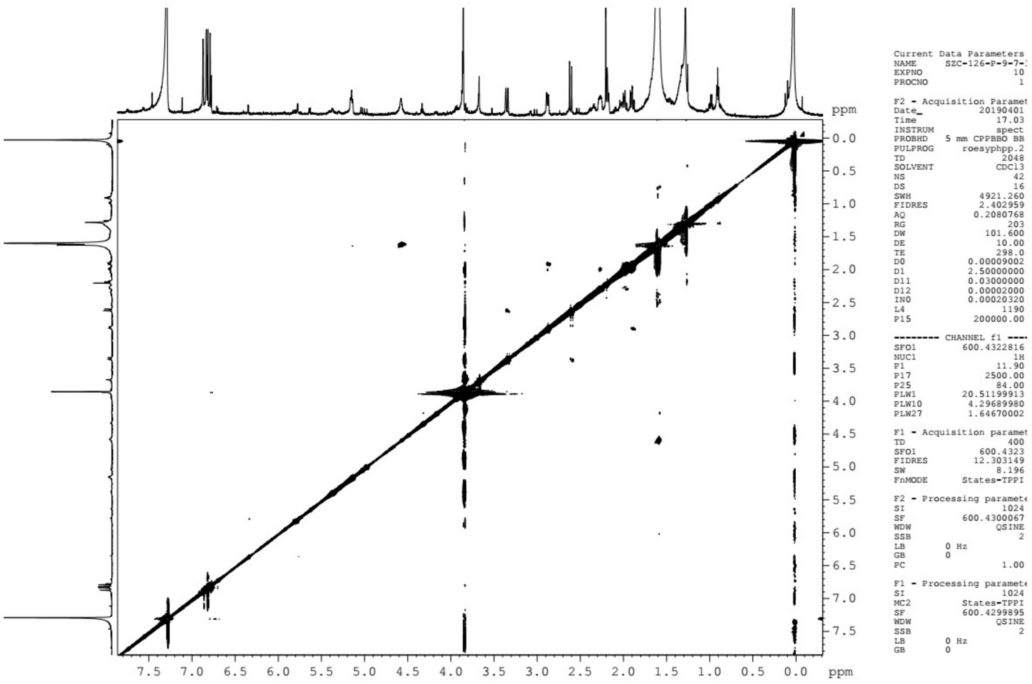

Figure S27. ROESY spectrum of the new compound **4**

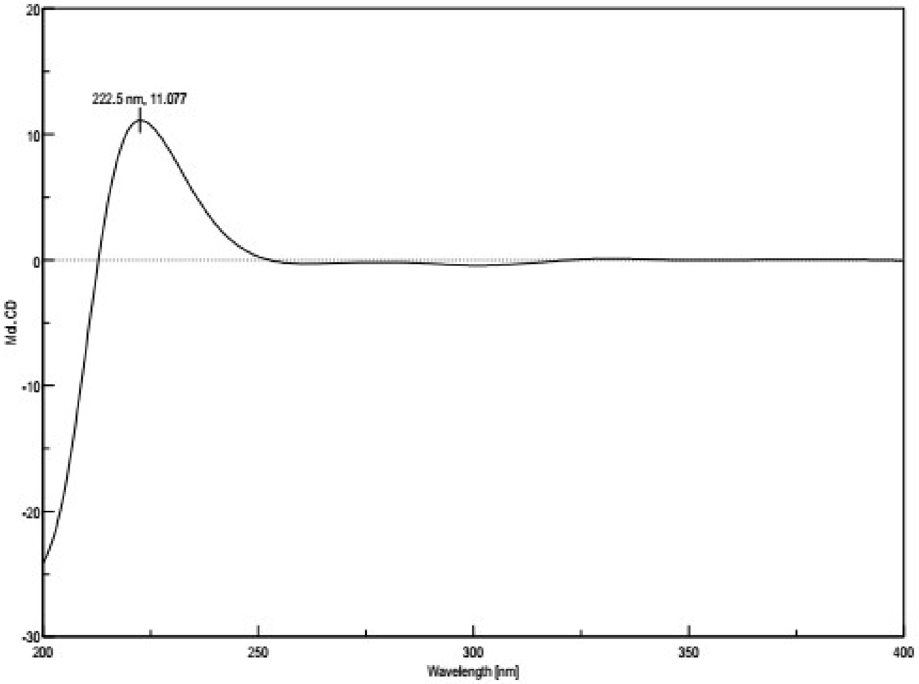

Figure S28. CD spectrum of the new compound **4**

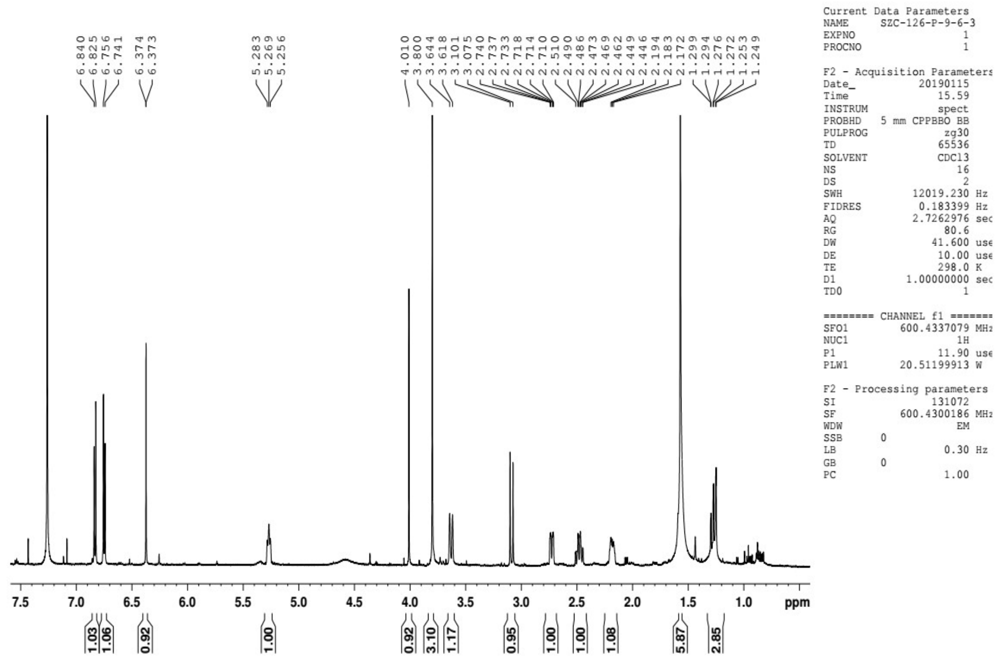

Figure S29. ^{1}H-NMR (600 MHz, $CDCl_3$) spectrum of the new compound **5**

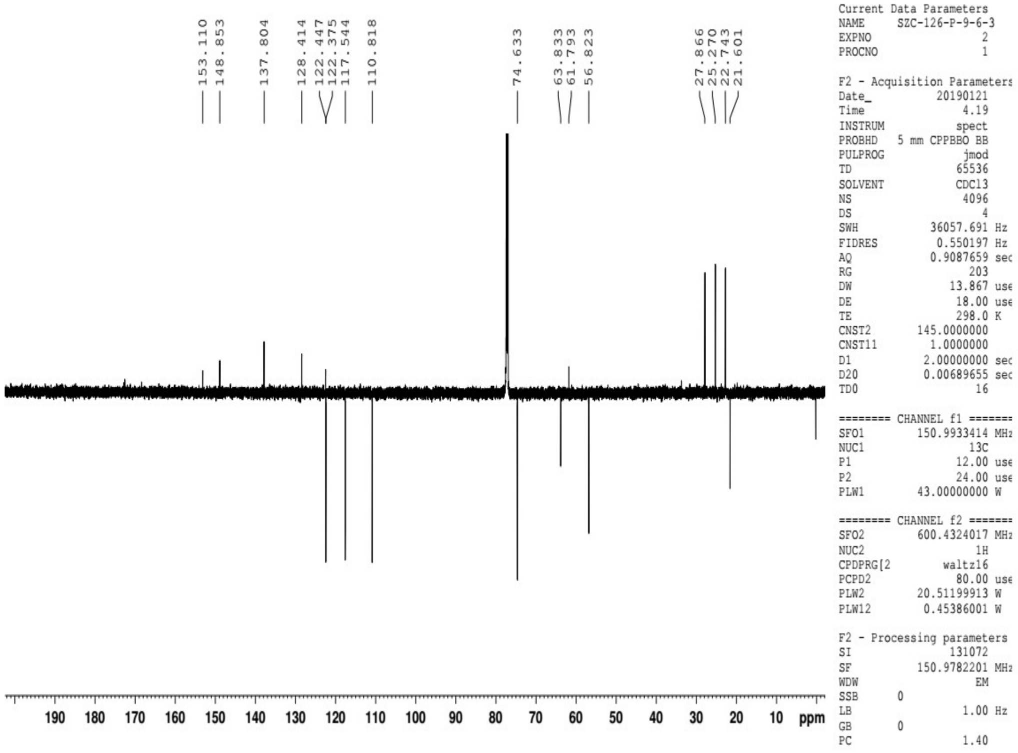

Figure S30. ^{13}C-APT (150 MHz, $CDCl_3$) spectrum of the new compound **5**

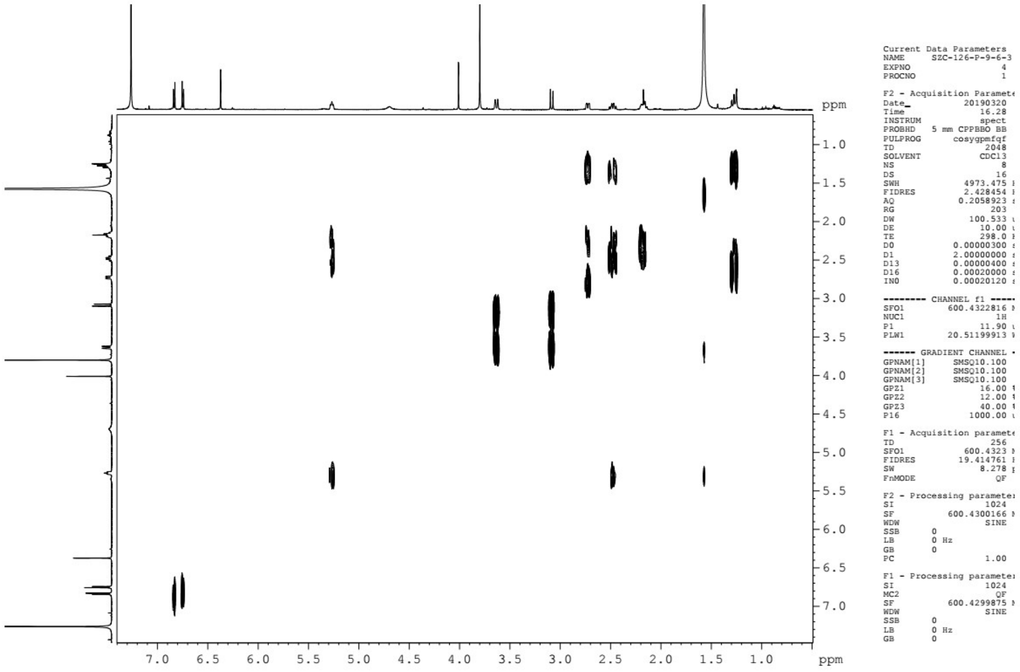

Figure S31. ^{1}H-^{1}H COSY spectrum of the new compound **5**

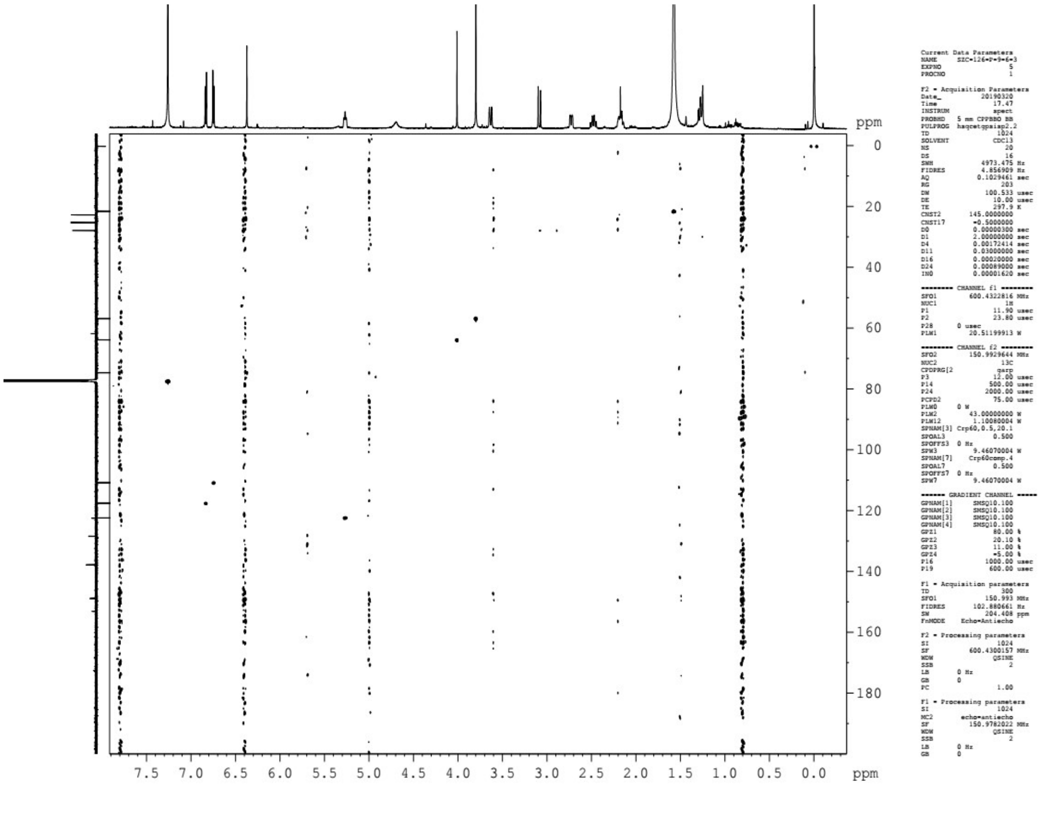

Figure S32. HSQC spectrum of the new compound **5**

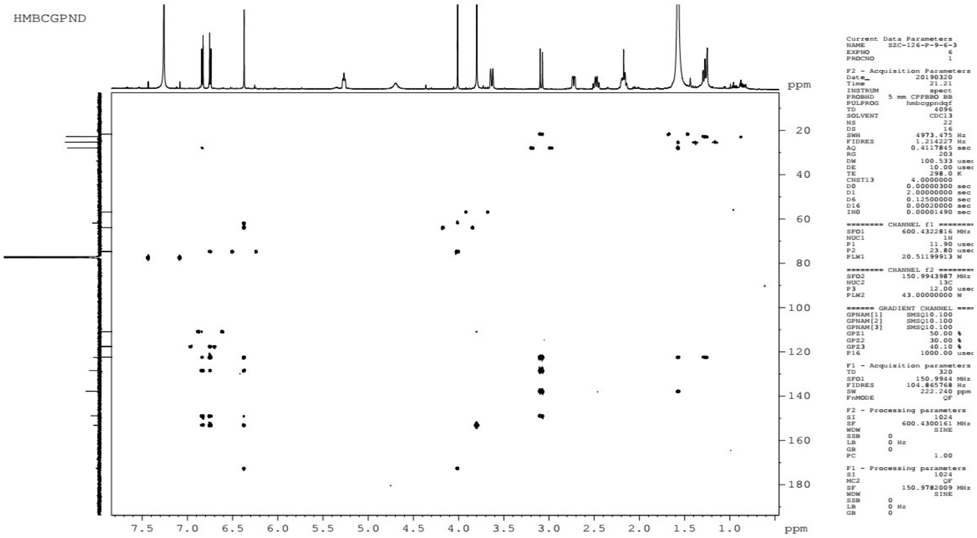

Figure S33. HMBC spectrum of the new compound **5**

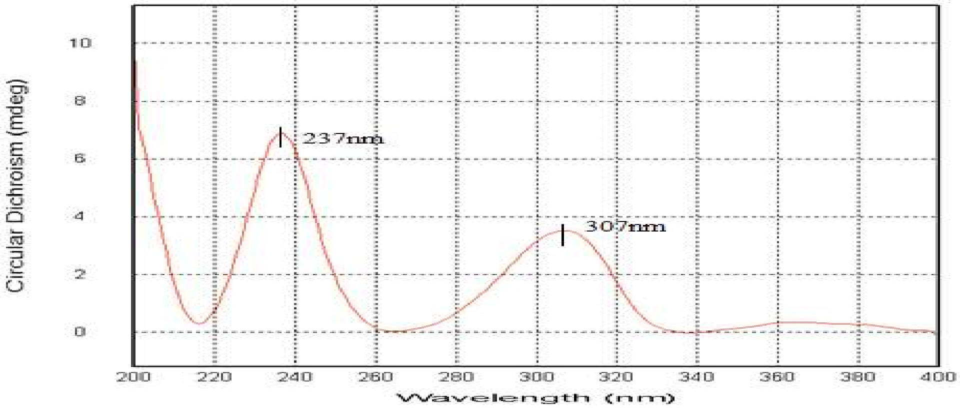

Figure S34. CD spectrum of the new compound **5**

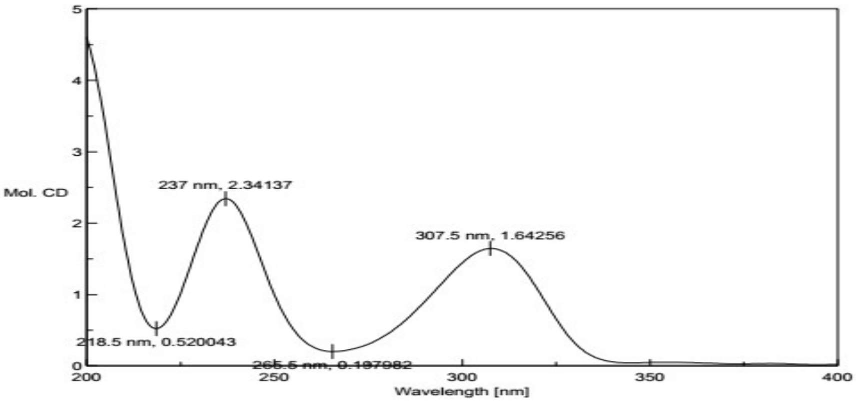

Figure S35. CD spectrum of clavilactone A
